# Oral cleft prevention program (OCPP)

**DOI:** 10.1186/1471-2431-12-184

**Published:** 2012-11-26

**Authors:** George L Wehby, Norman Goco, Danilo Moretti-Ferreira, Temis Felix, Antonio Richieri-Costa, Carla Padovani, Fernanda Queiros, Camilla Vila Nova Guimaraes, Rui Pereira, Steve Litavecz, Tyler Hartwell, Hrishikesh Chakraborty, Lorette Javois, Jeffrey C Murray

**Affiliations:** 1 University of Iowa, Iowa City, IA, USA; 2 Genetic Counseling Service – Bioscience Institute, São Paulo State University, Botucatu, Sao Paulo, Brazil; 3 Hospital de Reabilitação de Anomalias Craniofaciais, Bauru, Sao Paulo, Brazil; 4 Hospital de Clínicas de Porto Alegre, Porto Alegre, Rio Grande do Sul, Brazil; 5 Hospital Santo Antônio- Centrinho: Obras Sociais Irmã Dulce, Salvador, Bahia, Brazil; 6 Instituto Materno Infantil Prof. Fernando Figueira, Recife, Pernambuco, Brazil; 7 RTI International, Durham, NC, USA; 8 Eunice Kennedy Shriver National Institute of Child Health and Human Development, Bethesda, MD, USA

**Keywords:** Oral clefts, Cleft lip, Cleft palate, Craniofacial anomalies, Congenital anomalies, Birth defects, Folic acid, Vitamins, Prevention

## Abstract

**Background:**

Oral clefts are one of the most common birth defects with significant medical, psychosocial, and economic ramifications. Oral clefts have a complex etiology with genetic and environmental risk factors. There are suggestive results for decreased risks of cleft occurrence and recurrence with folic acid supplements taken at preconception and during pregnancy with a stronger evidence for higher than lower doses in preventing recurrence. Yet previous studies have suffered from considerable design limitations particularly non-randomization into treatment. There is also well-documented effectiveness for folic acid in preventing neural tube defect occurrence at 0.4 mg and recurrence with 4 mg. Given the substantial burden of clefting on the individual and the family and the supportive data for the effectiveness of folic acid supplementation as well as its low cost, a randomized clinical trial of the effectiveness of high versus low dose folic acid for prevention of cleft recurrence is warranted.

**Methods/design:**

This study will assess the effect of 4 mg and 0.4 mg doses of folic acid, taken on a daily basis during preconception and up to 3 months of pregnancy by women who are at risk of having a child with nonsyndromic cleft lip with/without palate (NSCL/P), on the recurrence of NSCL/P. The total sample will include about 6,000 women (that either have NSCL/P or that have at least one child with NSCL/P) randomly assigned to the 4 mg and the 0.4 mg folic acid study groups. The study will also compare the recurrence rates of NSCL/P in the total sample of subjects, as well as the two study groups (4mg, 0.4 mg) to that of a historical control group.

The study has been approved by IRBs (ethics committees) of all involved sites. Results will be disseminated through publications and presentations at scientific meetings.

**Discussion:**

The costs related to oral clefts are high, including long term psychological and socio-economic effects. This study provides an opportunity for huge savings in not only money but the overall quality of life. This may help establish more specific clinical guidelines for oral cleft prevention so that the intervention can be better tailored for at-risk women.

**ClinicalTrials.gov Identifier:**

NCT00397917

## Background

### Study objectives and significance

#### Statement of the problem

Craniofacial anomalies comprise a significant component of morbid human birth defects. They require surgical, nutritional, dental, speech, medical, and behavioral interventions and impose a substantial economic burden. Clefts of the lip and palate affect about 1/700 births with wide variability related to geographic origin [1] and socioeconomic status [2]. The complex etiology of cleft lip with or without cleft palate (oral clefts) affords ample opportunities to identify environmental and gene-environment interactions and to establish programs for prevention.

Numerous studies have looked at health inequalities and group differences on worldwide populations, often under the directorate of the World Health Organization (WHO) [3,4]. Mechanisms for measuring the burden of these diseases are controversial, but some standard methodologies are beginning to emerge [5]. Studies in Latin America suggest that health expenditures in total are currently less than one-tenth per capita of what they are in developed nations [6]. As countries become more developed, these expenditures will increase and begin to address complex problems such as birth defects, which currently remain largely untreated and ignored in the indigent populations of South America. Although at the present time, birth defects, and cleft lip and palate in particular, are not substantial contributors to the overall global burden of disease [7], it is clear from predictive models that over the next 20 years birth defects will play an increasingly important role in the burden of disease. Birth defects will eventually supplant infectious diseases and prematurity as a single major cause of morbidity and mortality in the first month of life, as has become evident in Western developed populations.

An effect of vitamin supplementation on the incidence of cleft lip and palate has been hypothesized for over 40 years [8]. A number of subsequent studies reviewed by Czeizel [9] and Munger [10] have continued to suggest that folic acid and/or other micronutrients or vitamins, including vitamin A and vitamin B6, may be important in the etiology of clefts as well. These studies strongly support further investigations of the role of vitamins and other environmental components in clefting and compel the determination as to whether interventional strategies can result in decreases.

#### Genetics and epidemiology

CL/P affects about 1/700 births with wide variability across geographic origin [1] and socioeconomic status [2]. In general, Asian or Amerindian populations have the highest birth prevalence, often as high as 1/500, with European derived populations intermediate at about 1/1000, and African-derived populations the lowest at 1/2500. In South America, the incidence of CL/P is 1/1150 [11] and the recurrence rate among sibs is about 4%, similar to sibling risks in other populations [12].

CL/P is thought to result from a complex interplay of genetic and environmental factors. In humans, a finely choreographed cascade of gene expression, cell migration, cell transformation and apoptosis between 14 and 60 days post conception creates the soft and hard tissues of the face from the originating oropharyngeal membrane. By 48 days the upper lip is continuous and by 60 days palatal shelf fusion completes facial embryogenesis [13]. Disruption of any of the tightly regulated processes occurring in this time frame by environmental and/or genetic abnormalities may then predispose to cleft lip and/or palate. Clefts can be divided into nonsyndromic (NS) and syndromic oral clefts. In NS oral clefts, affected individuals have no other physical or developmental anomalies [14]. Most studies suggest that about 70% of cases are NS [15]. The remaining 30% of cases are considered syndromic. The etiology of syndromic cases includes chromosomal syndromes, Mendelian disorders, teratogens (e.g., phenytoin or alcohol), and uncategorized syndromes with more than 400 forms reported in Online Mendelian Inherited Diseases in Man (OMIM). Facial clefts also have been divided into those that affect the lip only or the lip and palate together from those that affect the palate only. This division is based on both genetic (recurrences are almost exclusively limited to a single group in first degree relatives) and embryologic (the anterior or hard palate forms separately from the posterior or soft palate). In this study, we will include non-syndromic CL/P cases only (i.e. clefts of the lip or the lip and palate together) to avoid the confounding effects of cleft palate only, recognizing that there may be overlap in etiology and recurrence prevention.

Fogh-Andersen [16] in Denmark first proposed genetic factors in clefting, which have subsequently been confirmed by segregation analysis [17] and twin studies [18]. The similarities in predicted mechanisms, genes and environmental contributions across populations including European/North American [17], Asian [2] and South American [19,20] make it likely that the effects of recurrence prevention observed in one region can be extrapolated to other regions. This is especially relevant for Brazil (where this study will take place) and the US, as there are varying degrees of admixture with Europeans, Native Americans and Africans [21] but significant overlaps with US populations. Bolstering this argument for cross ethnic similarities are data showing that NTD prevention by folic acid is similar in China, Chile and the US (see below). A few specific genetic contributors to cleft etiology have begun to be identified including TGFA, D4S192, MSX1, TGFB3, and RARA, and IRF6 [22-34] but the majority remain unexplained.

#### Environmental studies

An environmental component to clefting was recognized when Warkany [35] associated nutritional deficiencies with cleft palate [reviewed in Murray [14]]. Nutritional factors continue to be supported [36] as does low socioeconomic status [2]. Recognized teratogens that cause clefts include rare exposures, such as phenytoin, valproic acid and thalidomide, and also common environmental exposures, such as maternal alcohol or cigarette use and herbicides such as dioxin [37]. There is strong support for an increased risk of CL/P with maternal smoking [38-41]. Two meta-analyses have estimated increased odds by a factor of 1.3 for CL/P occurrence with maternal smoking [42,43]. Maternal alcohol consumption has also been reported to increase the risk of clefting [44-46]. Exposures to drugs such as anticonvulsants [47], Benzodiazepines [48], and corticosteroids [49,50] have also shown an increased risk for oral clefts.

Other epidemiological studies also support a role for environmental factors in clefting, especially in regions of low socioeconomic status (SES). In the Philippines, three studies [2,51,52] all report incidences of CL/P of 2/1000 in indigent populations while complementary studies show an incidence of 1.2/1000 in native Filipinos living in areas of higher SES, including Manila [52]; Hawaii [53] and California [54]. In South America, heterogeneity of birth defect rates also suggests environmental etiologies [55] with altitude noted as a particular risk factor [56]. Thus, nutritional or toxic environmental exposures may contribute directly to as much as one-third of cleft cases, and etiologies will be most identifiable and preventable in indigent populations.

#### Gene-environment interaction

The development of single nucleotide polymorphisms (SNPs) provides the opportunity to develop assays that are gene specific and often functionally relevant. Identification of SNPs is available both through genome wide efforts including the recently completed HapMap project [57]. Studies of gene-environment interactions can exploit advances in methods development [58] and for CL/P present some interesting data. Interaction between smoking and TGFA has been reported primarily for CPO [59-61], but not in other studies [45,62-64]. Interactions between smoking and MSX1, TGFB3, NAT1, NAT2, CYP1A1, GSTT1, GSTM1, and EPHX1 have all been studied [45,65-69], with some suggestive but generally modest effects.

Interactions between vitamin use and the folate metabolic pathway have also been intensively studied. Folate plays a pivotal role in DNA synthesis and methylation, and contributes to both development and gene expression. Metabolic forms of folate are involved in synthesis of nucleotides and in the methionine cycle, which generates methyl groups that are essential for DNA methylation. Genes that code for folate metabolizing enzymes, such as Methylenetetrahydrofolate reductase (MTHFR), are optimal candidates for gene-folic acid interaction studies given the suggestive results of the role of folic acid supplementation in incidence and recurrence of oral clefting. Specific alleles in these genes, such as the C677T of MTHFR, may modify the effects of folic acid supplementation. Genes that are good candidates for consideration include MTHFR, MTHFD, MTR, MTRR, RFC1, GCP2, CBS, BHMT, BHMT2 and TS.

There are numerous and often contradictory studies for the MTHFR C677T variant [33,70-73]. Changes in serum/red blood cell folate and homocysteine with increased dietary folate consumption after low folate consumption have been reported to vary by MTHFR 677 status [74]. A potential interaction between vitamin use and RFC1 has also been suggested [75,76], though no evidence has been observed in a recent study [77]. In summary, there is as yet little consensus among the many studies of interaction between vitamin/folic acid use and genetic factors in the etiology of CL/P.

#### Observational studies of multivitamin and folic acid use and clefts

Several case–control observational studies have reported a protective effect of periconceptional use of multivitamins and folic acid on occurrence of clefts [see Botto et al. [78] for a review]. The estimated decrease in CL/P risk with supplements containing folic acid has ranged from 18% [79] to 50% [80,81]. Studies of multivitamin use without specification of folic acid content have reported risk reductions of 30% [82], 40% [83,84], and 50% [85]. The smaller risk reductions were generally not statistically significant. Only one observational study [86] has reported an increased risk for CL/P with folic acid supplements (by 30% but insignificant) yet their control group included only children with birth defects outside of midline defects, which might be interpreted as a protective severity reduction effect of folic acid for those anomalies.

Other studies of micronutrient and folate exposures have also suggested associations with oral clefts in humans. Dietary folate intake was reported to be associated with oral cleft risk by up to 70% increase with 0.18mg compared to 0.35mg of daily folate intake [87]. Low maternal B6 [10] measured after pregnancy was reported to increase the risk of CL/P, particularly in cases with low serum folate. Post pregnancy low B12 levels and low infant serum folate have also been linked to increased oral cleft risk [81]. B1 and B6 deficiencies in maternal diet were also associated with an increased risk of oral clefts [88] as were myo-inositol and zinc [89,90].

Exposure to folic acid antagonists such as antiepileptic drugs and dihydrofolate reductase inhibitors was reported to double the odds for oral clefts [91]. Animal studies also provide support for anti-teratogenic effects of prenatal folic acid supplementation and dietary folate. Peer et al. [92] showed a 69% reduction in cortisone induced cleft palate occurrence in mice with folic acid injection; there was an 82% reduction with a combined treatment of folic acid and B6. Folic acid supplementation was also shown to decrease the frequency of retinoic acid induced cleft palate in mice by up to 92%, with suggested additive effects with methionine [93]. Procarbazine induced cleft palate was also reported to decrease with folic acid supplementation in rats [94-96] with potential dose and gender dependent effects of folic acid. Supplementing mice who have a higher risk of spontaneously occurring oral clefts (A/WySn Mice) with folinic acid, a metabolic form of folic acid, was reported to decrease the frequency of CL/P by up to 75% [97]. Adequate dietary folate decreased the teratogenic effects of methanol in mice by about 74% [98], and very low folic acid diets have been shown to delay the secondary palate closure in mice [99]. Supplementation with 5 mg/day dose of folic acid was reported to decrease the occurrence of cleft palate in dogs by 76% [100]. These studies also provide suggestive results for a potential role of folic acid and possibly other micronutrients in oral cleft etiology/prevention.

#### Folic acid fortification and oral clefts

A few countries have introduced folic acid fortification of grain and flour given the strong evidence for a preventive effect of folic acid on neural tube defects (NTDs). Indeed, this evidence and its subsequent application to populations is one of the major public health successes in the field of birth defects (see below). Unlike the case for NTDs, there is no converging evidence for significant changes in birth prevalence for oral clefts post folic acid fortification. In the United States, where folic acid fortification of grain products was mandated on January 1, 1998, three studies have generally found non-significant reductions of 3% [101], 5% [102], and 12% [103] in CL/P prevalence post fortification. A significant 12% reduction in CPO prevalence has been reported [102].

A slight non-significant increase in the prevalence of oral clefts has been reported after two years of fortification of cereal grain products (1998 through 2000) compared to pre-fortification (1994–1997) period in Ontario, Canada [104]. Also, in a preliminary evaluation of the effects of fortifying wheat flour with folic acid in Chile starting 2000, Castilla et al. [105] reported no significant changes in prevalence of oral clefts through the end of 2001 compared to 1999, while significant change of 31% was shown for NTDs. Longer periods may be required for a more comprehensive evaluation of potential changes in prevalence of oral clefts post fortification, yet given the evidence of NTD reduction of up to 50% in similar periods [e.g. [102,106-108], see Mills and Signore [109] for a review], these results suggest that low doses of folic acid may be inadequate to even prevent primary occurrence of oral clefts. Further, these studies of prevalence changes over time also suffer from limitations including potential confounding by other simultaneously changing contributory factors and the lack of well-matched control groups.

In Brazil, fortification of wheat and corn flour with folic acid became mandatory in June 2004. Flour is fortified at a dose of 150 μg/100 g, which is lower than that used in Chile (220 μg/100 g of flour) and comparable to the dose used in the United States (average of 150 μg/100 g of cereal grain product). Assuming that women consume about 200 g of fortified flour on average per day, the daily intake of folic acid would be expected to increase by about 300 μg.

#### Interventional studies of folic acid and neural tube defects (NTDs)

Several clinical trials have evaluated the effectiveness of folic acid supplementation at high or low doses in prevention of NTDs. These studies have provided strong evidence for a large preventive effect of folic acid on both recurrence and occurrence of NTDs. The strongest evidence for a preventive effect of high dose folic acid supplementation on recurrence of NTDs comes from the Medical Research Councial (MRC) 1991 double-blinded randomized study, where women with a previous child with NTD were randomly assigned to groups of 4 mg folic acid, other vitamins, vitamins with 4 mg folic acid, and placebo, taken daily at preconception and throughout the first trimester of pregnancy. The folic acid groups had a lower relative risk of NTDs in offspring of 0.28 compared to the other groups, indicating a 78% decrease in recurrence risk of NTDs. No significant decreases in NTD recurrence were observed in the group receiving other vitamins. This indicates that preventive effects are due the folic acid component, though the study was not powered enough to detect potential interactive effects between folic acid and the other vitamins.

Multivitamin supplementation with a 0.8 mg folic acid at preconception and through at least two months post conception was also shown to lower the risk of first occurrence of NTDs by up to 100% in a randomized clinical trial in Hungary using a sample of women with no history of NTD in offspring [110]. This same study showed no decrease in the occurrence of CL/P though the overall rate of non-NTD other genetic syndromes was also reported to decrease. As a confirmatory study applying a two-cohort controlled design in Hungary, with the interventional group receiving the same folic acid containing multivitamin as Czeizel [110] also found a significant decrease in NTD occurrence by up to 89% and in cardiovascular defects (40%), but no significant decrease for oral clefts.

Intake of 0.4 mg folic acid beginning before conception and continuing in the first trimester of pregnancy was shown [111] to decrease the occurrence of NTDs in China by up to 79 percent in a sample from the northern area with higher baseline rates of NTDs (5/1000 births) and by 16 percent in the Southern region sample (baseline rates of 1 per 1000). In the groups with >80% compliance with folic acid, there were 85% and 41% reductions in NTD occurrence in the Northern and Southern samples respectively. These results strongly indicate that the preventive effects on recurrence and occurrence of NTDs are due to the folic acid component rather than the other vitamins, though interactive effects have not been thoroughly evaluated. The NTD research provides a model for developing clinical trials aimed at assessing preventive effects of folic acid on recurrence and occurrence of oral clefts of direct relevance for clinical practice.

A connection between NTDs and CL/P can be supported by their similar time of occurrence during embryogenesis, their status as defects involving the midline of the embryo, their near identical population genetic characteristics (variable by geographic origin but with near identical recurrence risks and very similar birth prevalances overall), evidence of similar gene/environment contributions and the failure to identify any major genetic factor for either. The mechanisms by which folic acid might prevent NTDs or other birth defects remain unexplained. It might be secondary to the need to overcome pharmacogenetic deficiencies in women who require higher baseline intakes to reach therapeutic levels. A recently proposed mechanism relates to antibodies to the folic acid receptor [112]. The role of antibodies to the folate receptor has yet to be confirmed but could explain why some women respond to high doses of folic acid as this may be required to titer the effects of antibody bound to receptors. The pharmacologic rescue by high dose folic acid has been reported in a rat model where folate receptor antibodies induced intracellular folate deficiency associated with birth defects [113].

#### Interventional studies of folic acid and oral cleft recurrence

Only a handful of interventional studies have been conducted over the last 48 years to study the effect of folic acid supplementation on recurrence of oral clefts in mothers with a child with CL/P. The decrease in cleft recurrence among the folic acid groups reported in these studies, independent of statistical significance, ranges from 24 to 100% and is summarized in Table 1.

**Table 1:** Summary of non-randomized interventional human trials for cleft recurrence*

**Study**	**Intervention**	**Supplemented group**		**Control group**		**Reduction**
		**# births**	**RR (%)**	**# births**	**RR (%)**	
Conway (1958)	MV and 0.5mg FA	59	0.0	78	6.4	100% (p=0.1)
Peer et al. (1964)	MV and 5mg FA and 10mg B6	176	2.2	418	4.7	53% (p=0.1)
Briggs 1976 [extension of Peer et al. (1964)]	MV and 5mg FA and 10mg B6	228	3.1	417	4.8	35% (p=0.2)
Tolarova (1982)	MV and 10mg FA	84	1.2	206	7.3	84% (p=0.02)
Tolarova and Harris (1995)	MV and 10mg FA	211	1.4	1824	4.2	66% (p=0.03)
Total**		*498*	*2.01*	*2319*	*4.38*	*54.1%*

MV=multivitamin; FA=Folic acid; RR=Recurrence Rate.

* Conway [8], Peer et al. [114] and Briggs [115] included mothers to children with cleft lip and/or cleft palate. Tolarova [116] included mothers to children with cleft lip with/without cleft palate (CL/P). Tolarova and Harris [117] included women with CL/P or mothers to a child with CL/P.

** The total recurrence for treated and control groups is based on total number of affected births divided by total group size (or proportion of each study’s recurrence relative to its sample size). Peer et al. [114] and Tolarova [116] are excluded as these data were included in other studies.

Conway [8] reported no recurrent cleft cases among 59 births to mothers with history of cleft lip and/or cleft palate in previous births. They received a multivitamin that included 0.5 mg of folic acid and the recurrence rate in a group of 78 births to mothers who did not receive the supplement was 5.1%. Peer et al. [114] reported a 53% reduction in the recurrence of cleft lip and/or cleft palate in a group of 176 women who received a multivitamin in addition 5 mg folic acid and 10 mg vitamin B6 during the first pregnancy trimester, compared to a control group of 418 mothers (p=0.1). In an extended study of Peer et al. [114] with more supplemented women, Briggs [115] reported a 35% reduction in recurrence of cleft lip and/or cleft palate (p=0.2), but a 65% reduction in CL/P recurrence (p=0.06). Tolarova [116] reported an 84% reduction in recurrence of CL/P in a group of 80 women who received a multivitamin and 10 mg of folic acid during three months before conception and throughout the first three months of pregnancy (p=0.02), compared to a control group of 202 women. Using data on a larger sample that included women with CL/P (40% of intervened sample) and mothers of a child with CL/P, and the same intervention as Tolarova [116] and Tolarova and Harris [117] reported a 66% reduction in recurrence of CL/P (p=0.03) The average reduction effect when combining the unique data samples of these studies [8,115,117], is 54.1% (47% when Conway (1958) is excluded), and 66.5% for CL/P recurrence combining Tolarova and Harris [117] and the CL/P sample of Briggs [115]. These calculations are inappropriate from a confirmatory side given the array of interventions and populations used, but from an exploratory perspective, may be helpful for gauging expected treatment effects of folic acid to form hypotheses in clinical trials. Our study hypothesis of 50% reduction is based on this result, the case–control observational study results, many of which also suggested similar effects, and the result of MRC study which showed a 78% in recurrence of NTDs [118].

The results of these studies are suggestive of potential preventive effects of high dose folic acid on cleft recurrence. The data from the Hungarian trials [79,110] also support the notion of lack of preventive effects of low doses of folic acid on occurrence of oral clefts. The NTD model showing preventive effects of high and low dose folic acid on recurrence and occurrence respectively, and the suggestive results from interventional studies and observational studies [119] for preventive effects of high doses on recurrence and occurrence of oral clefts strongly indicate that large doses of folic acid are best suited for evaluation in randomized clinical trials of recurrence.

While many of these studies used different doses of folic acid than what we are using, did not have a control group that also received 0.4 mg, and in some cases also included other multivitamins in the supplementation, the possible reduction in clefting rates nonetheless spans the range which we are attempting to demonstrate in this intervention. Other micronutrients could also be considered (B6, Zinc) but the case for folic acid alone is far more compelling.

#### Objectives and significance of proposed study

The studies reviewed above are suggestive of protective effects of folic acid supplementation on cleft risks, but all suffered from data and design limitations. The interventional studies for human recurrence have serious flaws, particularly in lacking a real control group generated by a randomization and in using combined interventions that do not allow for isolating the effects of folic acid [120]. Control subjects in these studies were usually women who either refused or did not comply with the intervention [116,117] or are otherwise poorly defined. This increases the chance of biases due to self or researcher selection of treatment, which confound the study results and introduces outcome differences that are not necessarily generated by the intervention. The different supplements and folic acid dose combinations make it hard to compare results and to attribute effects specifically. The sample sizes employed in these studies were also small and overall only borderline statistical significance was observed.

Observational case–control studies also have inherent problems, including non-random self or provider selection of supplement use. This is in part determined by perceived health risks that may also affect the risk for clefts. For example, women with unfavorable pregnancy histories, health problems or a family history of birth defects may use more folic acid but may also have a greater risk of poor pregnancy outcomes including CL/P. Further confounding results from the lack of data on health behaviors that may be correlated with both supplement use and oral cleft occurrence. Other limitations include potential bias in self-reported use of supplements and the lack of a well defined regimen for vitamin content, dose, and time of use. While helpful for exploratory research purposes, observational studies are not sufficient for answering the research hypothesis posed in this study.

Only a double-blinded, randomized experimental study, with sufficient sample sizes, can provide the opportunity to clearly address our hypothesis. The objective of this study is to assess the effect of folic acid supplementation of 4 mg/day taken at preconception and throughout the first three months of pregnancy on reducing the recurrence of CL/P compared to a 0.4 mg/day standard dose among women born with CL/P or mothers of children with CL/P using a double blinded randomized clinical trial design.

This study has the ability to identify, for the first time, the true preventive effects of folic acid on recurrence of oral clefts, using a dose that has proved effective in preventing recurrences of NTDs [118]. The double blind randomized design will cleanly separate the effect of the intervention from those confounding effects that have plagued previous interventional and observational studies. This study provides the chance to evaluate a treatment that if successful, will determine the standard of care for high risk women in the United States and abroad.

## Methods/design

### Study design

#### Objectives

The overall goal of this randomized, double-blinded study is to reduce the recurrence of non-syndromic cleft lip with or without cleft palate (NSCL/P) in a high-risk group of women supplemented with folic acid from preconception and continuing throughout the first three months of pregnancy.

#### Design

Subjects will be randomly assigned to 4 mg or 0.4 mg pills of folic acid (identical in appearance). A single pill of 4 mg or 0.4 mg of folic acid will be taken daily until pregnancy is documented. Once pregnancy is documented, the trial drug will be continued until 3 months after the last menstrual period. At this point, the trial drug will be discontinued and women will take routine prenatal vitamins until delivery following routine obstetric care. At the end of the study, recurrence will be assessed and compared between groups. The total sample recurrence rate as well as recurrence rates that are specific to the 0.4 mg and 4 mg folic acid groups will also be compared to the recurrence rates of oral clefts in a historical control group.

#### Primary hypothesis/primary outcome

The primary hypothesis is that folic acid supplementation of 4 mg/day at preconception and during the first three months of pregnancy will decrease the recurrence of NSCL/P in a high risk (history of NSCL/P in mother or child) group of women when compared to women taking 0.4 mg per day of folic acid. The primary outcome measure will be the difference in NSCL/P recurrence rates between the two groups and the associated confidence interval. The study is powered to detect a 50% reduction at a 0.8 power.

#### Secondary hypotheses

The following secondary hypotheses will be evaluated:

An increase in serum and/or red cell folate levels in all women following daily supplementation with folic acid.

When compared to the 0.4 mg supplemented groups, the 4 mg per day group will have the following:

‐Greater increases in serum and/or red cell folate.

‐Decreases in the recurrence of NSCL/P in each of the following subgroups:

•Women with NSCL/P themselves

•Women with one or more children with NSCL/P

‐A decrease in the severity of NSCL/P in offspring of trial mothers.

‐No increase in twinning rates.

No increase in miscarriage rates.

No increase in rates of preeclampsia.

‐No increase in rates of other birth defects.

‐No decrease in birth weight

‐No decrease in gestational age

A decrease in recurrence of NSCL/P in the group of study subjects compared to an historical control group obtained from the populations served by the craniofacial clinics participating in the study.

A greater decrease in the recurrence of NSCL/P in the group of study subjects receiving 4 mg of folic acid when compared to the historical control group relative to that of the group receiving 0.4 mg when compared to the same control group.

Under separate funding we will examine the role of genes involved in folate metabolism on folate, B12 and homocysteine levels, as well as potential interaction between these genes and the supplementation dose. This project will also establish a resource to examine the long-term outcomes of infants exposed to high dose folic acid in utero.

#### Historical control group

The recurrence rates in this study will be compared to that of an historical control group. The rationale for using this group relates to the inability to use a placebo control in this study due to ethical considerations. A comparison with an historical control group will allow the assessment of the combined effect of a minimum dose of folic acid of 0.4 mg and a maximum dose of 4 mg by comparing the NSCL/P recurrence rate in the total study group to that of a group of women who did not receive the folic acid dose of 0.4 mg per day. The effect of each of the two folic acid doses on the recurrence risk of NSCL/P could also be evaluated by comparing the NSCL/P rate in each of the two study groups (0.4 mg and 4 mg folic acid) to that of the historical control group.

The historical control will include women with NSCL/P or mothers of children with NSCL/P who are registered at the craniofacial clinics enrolled in the study. Those clinics have been involved in the provision of services to subjects with clefts for an extensive period, thus rates of cleft recurrence can be calculated over an extended time course providing not only cross-sectional data but also trends data adding to the validity of the historical control approach. Such data will reveal any changes in recurrence rates that might have occurred over time.

Taking into consideration the traditional internal validity challenges associated with using historical controls, no major historical events that could affect recurrence rates of NSCL/P in this population are expected to have occurred during the years immediately preceding this study.

Since the two randomized study groups (0.4 mg and 4 mg folic acid) will be compared to the same group of historical controls, any differences in the relative decreases of NSCL/P recurrence rates that may be observed between each of the study groups and the historical control group can be safely attributed to the effect of administered folic acid.

#### Pilot studies

##### Initial pilot study and subsequent recruitment at Bauru

With funding from NICHD’s Global Network for Women’s and Children’s Health Research, study enrollment was initiated in Bauru in January 2004. This initial pilot aimed at first recruiting about 500 subjects, identified through the records of the Hospital de Reabilitação de Anomalias Craniofaciais (HRAC) clinic, and located within 100 Km of Bauru, using a field-based strategy for recruitment and follow-up. Letters were mailed to 526 women inviting them to enrollment meetings in local communities; 181 women attended the enrollment meetings, and 134 women were enrolled (96% of those eligible). Several factors contributed to low attendance at these enrollment meetings. We found that 23% of the 500 subjects had either moved out of the catchment area or could not be located and that 12% had had a tubal ligation. The inconvenient recruitment and follow up strategies required multiple visits and poor compliance with these was amplified by poverty.

An extension of this pilot study to re-contact potential subjects from the first sample who did not attend initial enrollment meetings was carried out in June, 2004. About 280 subjects were re-invited to participate in the study; 100 women attended enrollment meetings and about 70 of them were enrolled in the study. During this second phase, we used 30 social workers from local public health offices to assist in contacting and locating subjects. The overall rate of attendance at enrollment meetings among potential subjects was more than 50%, and the rate of enrollment among eligible subjects was more than 85%. After this first pilot phase, we introduced some changes into recruitment strategies, including more flexibility in recruitment timelines, adding more social workers, and increasing the number of attempts of contacting subjects prior to enrollment meetings. We also developed a practical step by step recruitment decision tree to accommodate most recruitment scenarios. In November 2004, a third phase of recruitment was conducted in areas outside Bauru, targeting about 1,100 subjects and 285 were enrolled.

As of November 30, 2006, about 355 subjects are actively participating at the Bauru site and taking folic acid pills. To date, there have been 62 pregnancies in the Bauru sample; 3 resulting in miscarriages, 49 delivered and 9 are ongoing.

##### Revision of subject recruitment/follow-Up strategies and pilot at clinic sites

We identified some limitations in the strategies originally developed to identify, recruit, and follow up subjects at the Bauru site.

Inaccurate and outdated contact information with high population mobility decreased the number of available cases. 18% of potential subjects were untraceable.

Inviting subjects to attend prescheduled meetings for screening and enrollment lowered subject motivation to participate due to extra effort on their behalf, further intensified by the high prevalence of poverty in the targeted population.

The follow-up strategy also seemed intensive and burdensome by requiring subjects to attend follow-up visits and provide blood samples on a bimonthly basis, without providing any real direct incentives (besides potential benefits of the study pills). This also lowered compliance with the follow up schedule.

It became clear that the required sample size could not be secured through the Bauru site alone.

In order to address these limitations, we developed new sampling, recruitment, and follow up strategies using a clinic model. This strategy focuses on women who still attend the clinic for their own craniofacial care or for their children’s care, but can also include women who no longer attend the clinic regularly. We identified six new clinics in Brazil as candidates to join the study and pilot test these new strategies. Three of these clinics, Hospital de Clinicas de Porto Alegre (HCPA) in Porto Alegre, Hospital Santo Antônio- Centrinho-Obras Sociais Irmã Dulce (OSID) in Salvador, and Instituto Materno Infantil Prof. Fernando Figueira-CADEFI/IMIP (IMIP) in Recife formally joined the study. HCPA and OSID were able to start the pilot test studies. Recife was put on hold until additional funding could be secured.

The new recruitment strategies implemented at Porto Alegre and Salvador involve identifying potential subjects from subjects who attend the clinic for care. Registered subjects who are not scheduled to visit the clinic may still be invited by phone or mail to participate in the study. Initial screening and enrollment procedures occur as soon as a subject agrees to participate, often during first contact with subject, eliminating the need for extra visits for enrollment. Study pills are sent every two months to subjects by mail and health and pregnancy checkups are conducted mostly through phone rather than in person. In-person follow-ups are currently limited to the first two months after enrollment, to measure post supplementation folate and B12 levels, and every six months thereafter or at pregnancy. These procedures will be further eased in the proposed continuation. Food vouchers are being provided at enrollment and at every third completed follow-up.

This clinic-based strategy is expected to ensure a continuous increase in the number of study subjects and to enhance efficiency. Inaccuracies of contact information inherent in clinical registries can be avoided by this approach using in person contact. Further, the groups of subjects recruited through this approach may be more motivated to participate in prevention research given that the majority are still obtaining treatment either for themselves or their children and may thus perceive a higher burden of CL/P compared to groups with completed treatments. Reducing the frequency of blood collection and of in-person attendance by mailing pills and conducting follow up by phone also make subjects more willing to maintain active participation after enrollment. These strategies also require fewer personnel since most activities can be implemented at the clinic’s research unit by the clinical coordinators.

Subject recruitment began in May 2005 at Porto Alegre site. Through November 30 2006, 137 women have been screened and 103 enrolled. Recruitment activities began in Salvador in December 2005. Through November 30, 2006, 174 women have been screened and 106 enrolled. Recruitment and follow-up strategies have been successful with no significant problems. Mailing the pills and conducting follow-ups by telephone proved easy and effective.

### Study population and procedures

#### Study site and populations

The study will be conducted at four craniofacial sites in Brazil including Hospital de Reabilitação de Anomalias Craniofaciais (HRAC) in Bauru (state of Sao Paulo), Hospital Santo Antônio- Centrinho: Obras Sociais Irmã Dulce in Salvador (state of Bahia), Hospital de Clínicas de Porto Alegre (HCPA) in Porto Alegre (state of Rio Grande do Sul), and Instituto Materno Infantil Prof. Fernando Figueira-CADEFI/IMIP (IMIP) in Recife (State of Pernambuco). All of these clinics have had a long experience in providing care to patients with oral clefts.

#### Inclusion and exclusion criteria

##### Inclusion criteria

In this study, NSCL/P is defined as all cases of unilateral or bilateral clefts of the lip with or without cleft palate excluding the following: cases with recognized syndromes, cases with a chromosome abnormality, cases with one or more other major structural anomaly, cases with cognitive delay (IQ or equivalent less than 80), or cases exposed to recognized teratogens in utero (phenytoin or valproic acid). Cases with cleft palate only will not be included in the study as specified in Chapter 1. The following define the inclusion criteria for the study:

1. Women with NSCL/P, who are 16 to 45 years of age (after 45 fecundity decreases substantially) and who attend or have attended the craniofacial clinic for their care.

2. Women (age 16 to 45 years of age) who have at least one natural child of any age with NSCL/P who receives (received) care at the participating craniofacial clinics.

3. All women must reside in the catchment area of the study, which includes the state where the clinic is located and surrounding states.

##### Exclusion criteria

1. Having a first degree relative (that is a parent, sibling or child) who has cleft palate only.

2. Cases resulting from consanguineous couples (first, second, and third degree, i.e., first cousins or closer).

3. Couples where at least one of the two is definitely sterilized.

4. Women who are using intrauterine devices

5. Women who are using injectible contraceptives

6. Women on anti-epileptic drugs (The metabolism of anti-epileptic drugs requires a great deal of folic acid. Therefore, it is very difficult to verify the effect of folic acid on the prevention of clefting among these women. In order to avoid this situation, such women will not be included in this study).

7. Women on drugs that contain benzodiazepines

8. Women who are pregnant. They will be re-contacted later at an appropriate time for participation in the study.

9. Women who are planning to move outside of the catchment area of the study within the next year.

10. Women who have B12 deficiency (B12 level is below 174 pg/ml or 134.328 pmol/L).

11. Women who are allergic to folic acid.

#### Sampling, recruitment, and screening procedures

##### Sampling

Potential study subjects include a convenience sample of women with NSCL/P and/or mothers of at least one child with NSCL/P who currently attend or have attended in the past the study clinics for their own care or for the care of their children during the study period and who meet the inclusion criteria (age and residence) listed above. Potential subjects will be invited to participate. The majority of potential subjects (about 70%) are expected to be mothers of children with NSCL/P and the rest (30%) to be women who themselves have NSCL/P. Based on published literature and review of CL/P pedigrees at HRAC clinic in Bauru, the recurrence rates in the two subject groups of women with NSCL/P and mothers to a child with NSCL/P were estimated to be about 7 and 4 percent respectively.

While women up to 45 years of age are potentially eligible to participate, the sampling frame will be limited, when possible (e.g. when women’s age is known a priori from the clinic’s records), to women who are up to 40 years of age. The rationale for this sampling frame is that chances of pregnancy and propensities to have children decrease significantly after 40 years of age. Yet, due to potential inaccuracies in the age of women in the clinic’s records used for sampling, women who are invited to participate in the study and who are between 40 and 45 years of age will still be eligible to participate in the study on age basis in order to keep a positive environment for implementation of the study in the local community.

All potential subjects are identified from the clinics’ medical records and registries in addition to active surveillance of all patients who attend the clinics for care. The study staff will search patient records to identify potentially eligible subjects based on the primary information that are available in these records including CL/P diagnosis, women’s age, and residence. All patient charts from the past 10 years will be reviewed to identify women who themselves or their children have been treated at the clinic. Study staff will search the records of subjects who will attend the clinic to also identify potential subjects on a weekly basis. The study will be promoted to all the staff of these craniofacial clinics so that they also can assist in identifying and referring subjects to the study and will be advertised in local media.

##### Recruitment

Two models of recruitment will be applied:

1. ***Outreach model***: This model uses a field-based strategy to recruit potential subjects most of whom are no longer active patients at HRAC in Bauru. This model will be implemented by the Bauru study site, given the density of potential subjects in the proximate region who have been treated at HRAC. Field visits to conduct group recruitment meetings with potential subjects at local community facilities are the primary recruitment method in this model.

2. ***Clinic-based model*:** This model will use the craniofacial clinics as the sites for recruitment of subjects. Recruitment will be focused primarily on potential subjects attending the clinic during the study period. Additional recruitment activities will include identifying potential subjects from the clinic registry that are not active patients. This model will be implemented at all participating craniofacial clinics, including HRAC in Bauru.

##### Introduction to study and screening

Potential subjects will be contacted to verify the age and NSCL/P, present the study, and assess their interest to participate and willingness to be screened to determine final eligibility. Subjects who show interest will be offered to undergo immediate screening to confirm their eligibility.

##### Outreach model

The study staff will search patient records in the HRAC database to identify potentially eligible subjects based upon inclusion criteria in 3.2.1. The study staff will mail letters of invitation to identified potentially eligible subjects for attendance at prescheduled recruitment meetings. These meetings will take place at public facilities (e.g. health centers, schools, churches, or other public facilities) as coordinated with local community leaders. The study staff will follow-up with phone calls to check on intent to attend these meetings. In some cases, the local network of social workers may be used to visit invited subjects and confirm their intention to attend these meetings. This is especially helpful for contact with potential subjects with incorrect contact information in the HRAC database. During the first meeting, study staff will explain the purpose of the study, the study intervention, subject responsibility (e.g. compliance with study drug, maintaining follow-up), and design of recruitment, enrollment, and follow-up procedures. The study staff will offer to answer any questions the subject may have regarding the study, and will provide the subject with a study summary brochure. The study staff will screen those interested and enroll them if eligible.

##### Clinic-based model

The Clinical Coordinator will search patient records to identify a potential subjects that meet the inclusion criteria of NSCL/P and age, and will check the appointment schedule at the clinic to see if they may visit the clinic within the next six months. Subjects scheduled to visit the clinic in the next six months will be first contacted to participate in the study during their visit to the clinic. Potential subjects unscheduled to visit the clinic in the next six months and/or who no longer attend the clinic will be contacted via invitation letters and/or phone and invited to participate.

After verifying the potential eligibility status based on age and NSCL/P, the Clinical Coordinator will individually present the study to each potential subject. This first contact may take place at the clinic or via phone, and involves explaining the purpose of the study, the study intervention, subject responsibility (e.g. compliance with study drug, maintaining follow-up), and design of recruitment, enrollment, and follow-up procedures. The Clinical Coordinator will offer to answer any questions the subject may have regarding the study, and will provide the subject with a study summary brochure.

After presenting the study to the potential subject, the Clinical Coordinator will ask her whether she might be interested in participating in the study. Subjects who show interest will be offered to undergo immediate screening to confirm their eligibility. Subjects who show interest but are hesitant to be screened and enrolled will be given more time to consider their participation.

The Clinical Coordinator will ask subjects who are unwilling to participate about their reasons for not participating and whether any accommodations could be made to help them participate. This will be done in a non-coercive manner in order to inquire about information that may help to improve the study design and procedures. No further attempt will be made to recruit subjects who remain unwilling to participate.

#### Informed consent

Once screened, the Clinical Coordinator will provide the informed consent in the local language at the participating site to eligible subjects who are willing to be enrolled into the study. After reading the informed consent to the subject and answering any questions she may have, the Clinical Coordinator will ask her to sign the informed consent. If the subject is illiterate, confirmation will be obtained as a thumbprint in the presence of a witness. Women will be assured that refusal to participate in the study will in no way affect further treatment or care at the clinic.

#### Enrollment and baseline data collection

Once screened, the Clinical Coordinator will enroll eligible subjects who provide informed consent. The Clinical Coordinator will complete the enrollment form, and will obtain a blood sample (about 8 ml) from the subject for baseline measures of RBC, hemoglobin, B12, and serum/RBC folate.

Blood samples will be analyzed at a maximum of 2 weeks. Enrollment of subjects with normal B12 levels will be confirmed. Subjects found to be B12 deficient will be referred to a hematologist for treatment, and will be excluded from participation.

The Clinical Coordinator will randomize each confirmed subject into the 0.4 and the 4 mg folic acid groups (see section 3.6) and will dispense the folic acid pillbox with the relevant pillbox ID number. Study pills will be sent to randomized subjects by mail in most cases; subjects with unreliable addresses or who frequently attend the clinic for care receive their pills at the clinic. When delivered by mail, the clinical coordinator will contact the subject by phone to confirm their receipt of the pillbox.

#### Randomization procedures

Study subjects will be randomized to either 4mg or 0.4mg of folic acid. The study will be double-blinded; subjects, investigators and research staff will be blinded to the randomization assignments.

The Data Center will prepare the randomization sequence for the study using permuted blocks of random size. Randomization occurs at the subject level and is stratified by study site to ensure a balanced site representation in both treatment groups. The randomization will not be stratified according to risk group (mother with oral cleft or mother of child with oral cleft) or other factors due to the large sample size. As shown in [121], stratification becomes irrelevant after the sample rises above 200 subjects. Since randomization occurs at the mother’s level, the sample allocation should be very well balanced with respect to the risk group and other factors.

The randomization sequence will link the treatment assignment (0.4mg or 4mg) to a sequential list of serial numbers to be used for the study pill boxes. The Data Center will generate the randomization sequence. The Data Center will supervise the labeling of the boxes with the serial number. In addition, the boxes will be numbered in order of dispensing (1, 2, 3, etc.3). The serial and dispensing order numbers will be recorded on the study visit/follow-up form to track dispensing.

The random assignment will be accomplished by assigning the next serial number in the randomization list as the enrollment of each new subject is confirmed. Once confirmed, the study subject ID is recorded in a computerized randomization log (that will include randomization order, serial number of assigned pill box and the study subject ID). The Clinical Coordinators will affix the study subject ID labels to the boxes of each subject with the serial number assigned to her.

The Data Center will maintain the randomization sequence at RTI headquarters in NC. Revealing the individual assignment will be highly restricted and will only be done if deemed clinically necessary. Only a physician attending to the study subject may request revealing the treatment assignment for a clinical purpose. The Co-Principal Investigator will review this request and evaluate the necessity of revealing the treatment assignment for the given purpose. The individual assignment will be revealed only after the Co-Principal Investigator approves the request. The Manual of Operations details the procedures for requesting the individual assignment from the Data Center.

#### Study treatments

The study drugs are folic acid in concentrations of 4mg (high dose) and 0.4mg (low dose). The study drugs are supplied in tablet form, manufactured by ATIVUS Pharmaceutical Industries in Sao Paulo, Brazil. Quality control assessments of samples of production batches will be performed for all manufacturers in an independent quality control lab in the United States (Celsis Laboratory Group, Saint Louis, US). All pills, regardless of their folic acid concentration, will be identical in size, shape, and color and will be provided in identical packaging. Patients enrolled in the study will be instructed to ingest 1 tablet daily from enrollment until 3 months gestation.

The manufacturers will package the folic acid pills in boxes containing 5 blister packs of 14 pills each (2 rows of 7 pills), for a total of 70 pills per box. Each box will contain an information sheet that will include all recommendations of the country’s drug monitoring agencies (ANVISA for Brazil) regarding information about the drug, yet will not reveal in any way the actual folic acid content. The manufacturer will also label each of the pill boxes with the following information, in addition to any other item that is recommended by the monitoring agency:

Manufacturing and expiration dates (including only the month and the year of production which will be common for the two doses of folic acid)

Description of the drug (folic acid)

Identification of the manufacturer

Statement about the use of the drug (“this product is solely intended for research use and shall not be commercialized”)

Patient instructions for taking pills (or if a daily dose is missed)

Clearance number of monitoring agency

Clinic’s logo

The manufacturers will also label the blister packs with a production lot number. Each production run of pills will use several lot numbers for each dose. The lot numbers pertaining to each dose will be sent from the manufacturer directly to RTI after each production run.

After each production, RTI staff will supervise labeling the pillboxes with a serial number (##### - ##, a seven digit number with 5 digit base number and 2 check digits). A local staff independent of the study may be used to label the pill boxes.

There will not be any information on the pill box or blister pack that may indicate to the subject the folic acid content of the pills.

A sample of pills from the boxes will be selected from each production lot to be sent out for independent assay of folate content to assure manufacturing compliance.

#### Follow-up of study subjects

Subjects will be followed up every 2 months (approximately 8 weeks) post enrollment to deliver the study pills and evaluate their health status, pregnancy occurrence, and compliance with study pills. The study pills will be delivered to subjects through express mail when no in-person follow-ups are scheduled; subjects will be contacted by phone in this case to complete the follow-up data form and ensure that pills have been received. Periodic in-person follow-ups will be conducted every six months if subjects are available; otherwise a phone and mail follow-up will be conducted. During each follow-up, the subjects will be advised of the date and time of their next appointment. Within two weeks prior to each in-person follow-up, the Clinical Coordinator will contact the subject by mail or telephone to remind her of the follow-up date.

If subjects report menstruation delays of 14 days or more during the follow-up and pregnancy has not been confirmed, the study staff will advise the subject to take a pregnancy test either at the study clinic, at an outside clinic, or at home if subject prefers (subjects will be reimbursed for pregnancy tests outside of the study clinic).

Subjects who are confirmed to be pregnant will be advised as when to stop taking the study pill so that the intervention is limited to the first trimester of pregnancy. Gestational age will be calculated based on the last menstrual period. Pregnant subjects will be referred to prenatal care as soon as pregnancy is confirmed.

Pregnant subjects will be monitored by their local prenatal care providers. Participating subjects might receive care from various prenatal care providers during the pregnancy period and may not have a single prenatal care provider. Most women usually seek prenatal care at public health clinics, where they may get assigned a different provider at each visit. Thus it might be hard to maintain contact between the Clinical Coordinator and the prenatal care providers of the subjects. The Clinical Coordinator will attempt to initiate and maintain contact with identified prenatal care providers to follow up on pregnancy progress. The Clinical Coordinator will also maintain periodic direct contact through phone with the subject to check on her pregnancy progress.

Upon delivery, the Clinical Coordinator will follow with the subject and her physician to check on the occurrence of NSCL/P and other birth defects in the infant. Research staff may also access prenatal care records of subjects, pending approval of subject prenatal care providers, to abstract significant health and care events that occurred during pregnancy and that required physician care.

The Clinical Coordinator or Co-Prinicipal Investigator will also evaluate in person the infant for the presence clefting and other anomalies either through an appointment at the clinic or at subject’s home as convenient to the subject. When a birth defect is identified, research team members will carry out a complete physical exam and confirm all oral cleft diagnoses. The Co-Principal Investigator may also evaluate the infant along with the Clinical Coordinator or independently.

Gestational loss will be reviewed by one of the research team members (Clinical Coordinator, Co-Principal Investigator, and/or geneticists of the clinic). When available, miscarriage material will be examined. This examination will may include macroscopic analysis (presence or absence of NSCL/P; other apparent malformations), photographic documentation, and cytogenetic analysis in the event of malformations depending on the capacity of the clinic site in doing these investigations.

After completion of a pregnancy outcome, subjects will be asked about their willingness to resume participation in the study at an appropriate time following the end of pregnancy. Subjects who are willing to resume participation will be assigned to the same treatment group as their previous randomization assignment, and the standard enrollment procedures will be followed.

#### Blood collection

Folate (Serum and RBC) levels will be assessed, at baseline (prior to supplementation), every 12 months after enrollment, and at pregnancy. B12 levels will be assessed at baseline and subsequently every year to monitor for B12 deficiency. Folate and B12 tests may be conducted at the own laboratory facilities of the clinic/hospital when available or alternatively at local laboratories determined by the Co-Principal Investigator. The Clinical Coordinator and Co- Principal Investigator will monitor B12 levels and will refer subjects with B12 deficiency to the hematology service for treatment. Randomized subjects found to be B12 deficient may be temporarily or permanently suspended from the study drug based upon recommendations of the hematology referral.

Based on clinical and research experience, few subjects (perhaps none) are expected to ask for their blood results. If requested prior to the end of the study, an independent physician serving in the clinic will provide confidential results to the subject. The subject will also have the opportunity to discuss what her results mean (in terms of being within normal range or not) with the physician independently from other study subjects and research unit staff. This procedure will prevent sharing the results with other study participants as well as with any member of the research team. Since no data is available on what these folate measurements will actually mean in terms of the levels of folic acid supplementation that are provided, it would be impossible for any single woman to interpret these data points in a way that could result in her un-blinding. Therefore, any disclosure of folate test results under these specific circumstances will not affect the blinding of study subjects or of research unit personnel.

#### Management and retention of study subjects

All subjects will be provided with a toll-free phone number for the study that they could call at regular office hours or anytime in case of emergency for any questions they may have or issues they would like to report (pregnancy, illness, concerns, etc.).

The study will maintain periodic phone contacts with pregnant subjects to inquire on pregnancy progress and remain updated on subject’s residence. Pregnant subjects will be followed by their prenatal care provider. With subject approval, contact between the study staff and the prenatal care providers will allow follow up of the pregnancy and complications.

#### Incentives and reimbursements

Incentives, in the form of 30 Reais value food vouchers, will be provided to subjects participating in the study at enrollment and every six months thereafter to strengthen retention and motivation. All subjects will be reimbursed for transportation costs incurred to attend follow-up visits at the clinic. Postage costs for the returning of used pillboxes will be paid by the project.

As a substantial percentage of potential study subjects are economically disadvantaged, providing food vouchers is expected to help minimize the indirect burden of participation in the study, such as time lost from work or household production due to study requirements.

#### Protocol violations

A protocol violation must be reported to the Data Center within one week of its discovery. The Clinical Coordinator or the Co- Principal Investigator will complete the Protocol Violation Form. The Co-Principal Investigator will sign the form and submit it to the Data Center. Each protocol violation requires a completed form.

Possible protocol violations that require reporting to the Data Center include the following:

1. Informed consent was not obtained before randomization.

2. Subject did not meet inclusion criteria.

3. Subject did meet criteria requiring exclusion.

4. Study medication was never initiated for the patient.

5. Subject was permanently discontinued from study medication (e.g., early termination of study medication).

6. The wrong folic acid dose was given to the subject.

7. The treatment assignment of the subject was revealed to study personnel or to the subject either by request or inadvertent means.

8. Failure to take at least 50 pills in 2 months (A compliance rate of at least 80% is required).

9. Study subject withdrew consent.

10. The study subject became permanently lost to follow-up.

In situations where it is not clear whether a form is required, the Research Unit personnel should contact the Data Center staff to find out if a Protocol Violation Form is necessary.

If subjects are enrolled and later found to have been ineligible at the time of enrollment (violations 2 and 3, above), a decision to continue or terminate the study medication will be made depending on the particular inclusion/exclusion criteria violated as stated in the MOO. Except in specific situations listed in the MOO (such as when subject is permanently sterilized, has B12 deficiency and is recommended to be fully withdrawn from the study, takes epileptic drugs), subject’s enrollment will not be terminated in order to not violate the intention-to-treat principle. The medical status of a woman who discontinues the study medication will be monitored for 2 months following termination.

### Study interventions (Treatments)

#### Intervention (Treatment) descriptions

Eligible women with NSCL/P or with at least one child with NSCL/P will be randomly assigned to one of two treatment groups: one taking 4 mg and the other taking 0.4 mg of identically appearing folic acid pills. Subjects will take the folic acid preconceptionally and up to three months of gestation.

#### Delivery of interventions (Treatments)

An 8-week supply of study pills will be dispensed to subjects upon enrollment and at regular 8 week intervals to ensure uninterrupted dosing during the preconception and prenatal periods of participation. Study pills may be dispensed during in-person visits or delivered to subjects through express mail or in person visits to the clinic or subject’s home. In all sites, the pillbox provided for the previous supplementation period will be obtained from the subject to determine pill compliance.

Subjects who are confirmed to be pregnant will be advised as when to stop taking the study pills so that the intervention is limited to the first three months of gestation. If a subject experiences an adverse event, the study medication may be discontinued either temporarily or permanently after review of the principal or co-principal investigator and depending on the recommendation of the physician attending the subject when applicable.

#### Control group

The control group in this study will be the 0.4-mg folic acid group. A placebo control group is not used, as a low dose of folic acid is already recommended as a standard vitamin therapy for women during preconception and pregnancy period.

#### Related risks

Potential risks involved in this study include those of blood drawing and interview participation, which are felt to be minimal. Folic acid supplementation has overall three aspects that have already been discussed in the literature:

1. The use of folic acid may mask vitamin B12 deficiency in cases of pernicious anemia and cause irreversible neurological damage. In order to avoid this effect, a B12 assay on every subject will be performed prior to folic acid administration, and every year thereafter. Complete blood tests will be performed on collected samples at enrollment to help detect anemia. Current exposure to folic acid through fortification in the US has been reported not to increase the risk of masking anemia [122]. This does not imply though that high doses of folic acid do not necessarily increase the masking risk, mainly for cases with B12-deficiency related neuropathy with no anemia. Yet due to the small percentage of subjects expected to have B12 deficiency, the assessment of B12 levels on a yearly basis after supplementation provides an appropriate strategy to monitor subject safety related to this issue. The added cost compared to added benefit of more frequent blood sampling to measure B12 (or other analytes) is considered high in terms of increasing the burden on subjects and lowering motivation that it should be avoided.

2. It has been suggested in the literature, though not confirmed, that high folic acid doses, such as the one used in the present study, may increase the risk of spontaneous abortions (expected spontaneous abortion rate = 15%). The numbers of spontaneous abortions will be reported and the Data Safety Monitoring Board (DSMB) will determine whether these exceed the threshold for an adverse event.

3. It has been suggested in the literature, though not confirmed, that folic acid increases twinning. Should a pregnancy result in twins, twin pregnancies in general have an increased risk of premature delivery with an intendant risk of increased mortality and morbidity for the infant, and also an increased risk of maternal complications including pre-eclampsia. Therefore, information on this matter will be collected, and the DSMB will evaluate whether the rate of twinning exceeds the threshold for an adverse event.

### Measurement methods

#### Description of data forms

Table 2 describes the data forms that will be used for this study:

**Table 2:** Data forms

**Form #**	**Form title**	**Description**	**Completed by**
FA00	Sampling and Initial Recruitment Log Form	Documents the outcomes of searching the clinical records for potentially eligible subjects and of initial contacts with potentially eligible subjects including their interest in participation in the study.	Clinical Coordinator
FA01	Contact Form	Documents contact information for each potentially eligible subject and updated contact information for participating subjects.	Information extracted initially from clinical records and reviewed/updated with potentially eligible subjects and participating subjects by the Clinical Coordinator.
FA02	Screening Form	Documents eligibility for participation for each study subject selected.	Clinical Coordinator
FA03	Enrollment Form	Documents marital status, information about children, and smoking, alcohol use, menstruation, contraception and gynecological care of the study subject.	Clinical Coordinator
FA04	Enrollment Confirmation Form	Records blood test results, and study medication delivery to the subject, and plans for follow-up visit. This form may not need to be completed with the subject at some sites as all the information will be available to the Clinical Coordinator.	Clinical Coordinator.
FA05	Follow-up Form	Confirms address information, documents menstruation, administration of study medication, and plans for next follow-up visit.	Clinical Coordinator
FA06	Initial Prenatal Contact Form (With Study Subject)	Documents date of last menstruation, date of pregnancy confirmation, and information about the study subject’s doctor and source of prenatal care.	Clinical Coordinator
FA07	Prenatal Form (With Doctor)	Documents via periodic contacts with the subject prenatal care provider, or via abstraction from prenatal care records, information about the study subject’s pregnancy, prenatal vitamins, illness, ultrasound results, etc.	Co-Principal Investigator (initial contact),Clinical Coordinator
FA08	Study Continuation Post Miscarriage/Stillbirth Form (With Study Subject)	Collects data from the subject about her readiness to resume participation in the study after a miscarriage/stillbirth occurrence and arrange for the date and time of the next visit if she is willing to continue in the study.	Clinical Coordinator
FA09&10	Miscarriage/Stillbirth Form (With Doctor)	Documents the miscarriage or stillbirth date and age, as well assessment of miscarriage/still birth product if available including the presence or absence of clefting and other malformations, etc.	Co-Principal Investigator, Clinical Coordinator
FA11	Delivery Form (With Subject)	Documents date of delivery, birth weight and sex of the infant, mother’s smoking and drinking habits while pregnant, and any complications and malformations associated with the baby.	Clinical Coordinator
FA12	Delivery Form (With Doctor)	Documents any malformations that may be associated with the baby including craniofacial malformations and abnormal delivery events. Filled with the subject’s doctor (if possible) or abstraction from the medical records of the subject.	Clinical Coordinator
FA13	Phone Call Form	Documents telephone calls made to the study office by the study subjects, the reason for each call, and the action taken.	(Clinical Coordinator, Co-Principal Investigator)
FA14	Study Termination Form	Documents withdrawal from the study by a study subject, the reason(s) for termination and approval (or not) for a two-month post termination follow-up.	Clinical Coordinator
FA15	Post Termination Follow-up Form	Confirms address information, documents menstruation and whether or not the former study subject has gotten pregnant.	Clinical Coordinator
FA16	Laboratory Form	Documents the date of blood collection, date of analysis, and results of the blood testing.	Clinical Coordinator
FA17	Adverse Events Form	Documents all information related to any adverse event encountered during the course of the study.	Clinical Coordinator/Co-Principal Investigator
FA18	Protocol Violations Form	Documents all information related to any protocol violation encountered during the course of the study.	Clinical Coordinator/Co-Principal Investigator
FA19	Pregnancy Confirmation Form	Documents the results of a performed pregnancy test after randomization and related schedule of study folic acid supplementation	Clinical Coordinator
FA20	Prenatal Form (With Subject)	Documents via periodic contacts with the subject, information about pregnancy progress, prenatal vitamins, illness, ultrasound results, smoking/alcohol etc.	Clinical Coordinator
FA21 and FA21 Suppl-ement	Delivery Form (In-Person Evaluation of Live Birth	Documents the presence or absence of malformations and/or complications with birth upon in-person evaluation of the live born by the Clinical Coordinator.	Clinical Coordinator or Co-Principal Investigator and clinical geneticist (or pediatrician)
FA22 (Outreach-model only)	Pre-Contact Form	Documents the outcome of each attempt to contact subject by the study staff or voluntary professionals assisting on the study.	A study team member, Social worker, Parent Coordinator

#### Description of biological measures

The following laboratory measures will be collected from the subjects found in Table 3.

**Table 3:** Laboratory measures

**Laboratory measure**	**Timing**	**Aim**	**Performed by**
Complete blood count	At baseline, every 12 months post initiation of supplementation, and at pregnancy confirmation.	Anemia	Laboratory of study clinic
Pregnancy test	Whenever menstrual delay of 14 days or more is detected	Pregnancy	Laboratory of study clinic or an outside affiliated laboratory
Folate analysis	At baseline, every 12 months post initiation of supplementation, and at pregnancy confirmation.	Measure serum/red cell folate concentration	Laboratory of study clinic or an outside affiliated laboratory
Vitamin B12 analysis	At baseline and every 12 months thereafter	Detect pernicious anemia	Laboratory of study clinic or an outside affiliated laboratory

The following physical exam measures of babies born to subjects will be abstracted from medical records:

Birth defects

Birth weight

Length

Head circumference (when available)

The Clinical Coordinator will also obtain and document information on the presence of craniofacial malformations including clefts or other birth defects in babies born through personal evaluation of the infant, contact with the subject’s doctor after delivery, or abstraction from the medical record of the infant, but the clinical coordinator will make every attempt to evaluate in person every infant born into the study. Information about any malformations detected during the prenatal period through ultrasound testing will also be obtained from contact with the subject, her prenatal care provider, or through abstraction from the prenatal care record.

#### Schedule of data collection

Data will be collected during the various stages of subject sampling, screening, enrollment, follow-up, pregnancy occurrence and progress, and termination from study. General contact information (name, telephone, number, etc.) will be documented prior to introducing the potential subject to the study when possible. This information (telephone, residence, name, etc.) will be verified upon initial contact with the subject. Data will also be documented when screening willing subjects, which will occur either directly or within a week period after first contact with the subject. Data will also be collected during enrollment of eligible subjects, which will occur either directly or within a week period after screening, and during each bi-monthly follow-up. Upon occurrence of pregnancy, data will be collected through contact with subject and/or subject prenatal care provider and/or through abstraction from the medical record. The subject will be contacted at an appropriate time after pregnancy resolution, regardless of the pregnancy outcome, to obtain information on study outcomes and check on her willingness to resume participation in the study. Standard enrollment, data collection, and follow-up procedures will be followed with study subjects who agree to resume participation in the study. A study termination form will be completed with subjects who wish not to resume participation in the study, and a follow-up visit is conducted after two months, whenever possible, to check on pregnancy occurrence and source of prenatal care if subject is pregnant and obtain permission to contact her provider to inquire on her pregnancy.

Tables 4 and 5 below include schedules of data collection, procedures performed, data forms completed, and laboratory tests conducted before and after detection of a delay in menstruation respectively.

**Table 4:** Schedule A (Data collection before any detection of a menstruation delay)

**Task**	**Timing**	**Procedure**	**Forms**
Abstraction from clinical record	Sampling phase	Search clinical records to identify potentially eligible subjects	FA00
Introduction to study	First contact with subject (in person or by phone)	-Verify potential eligibility	FA00
		-Verify subject address and contact information	FA01
		-Introduce potential subject to the study	
		-Explain study purpose and subject responsibilities	
		-Answer any questions that potential subject may have	
		-Check willingness of potential subject to participate	
Screening	Directly or within a week period after introduction into study	Screen subject for eligibility	FA02
Enrollment	Directly or within a week period after screening	-Obtain informed consent	FA03
		-Obtain blood sample from subject	
		-Inform subject of process of study drug dispensing and next follow-up	
Verification of B12 and pregnancy test results, randomization, dispensing study pills, and baseline contact	Within 1–2 weeks days after enrollment	-Verify blood test results (mainly B12 levels)	FA04
		Randomize subjects who have no B12 deficiency and are not pregnant and dispense folic acid pills	
		Refer subjects who have B12 deficiency to hematology service	
Follow-up onward	Every 2 months (about 8 weeks)	-Dispense and confirm receiving folic acid pills	FA05
		-Complete follow-up visit form	
Follow-up when blood sampling is due	(Once every 12 months after enrollment.	-Schedule an in person follow-up for blood sampling	FA05
		-Dispense folic acid pills	FA16
		-Complete follow-up visit form	

**Table 5:** Schedule B (Data collection after detection of menstruation delay or pregnancy)

**Task**	**Timing**	**Procedure**	**Forms**
Verification of pregnancy and measuring folate levels (if pregnant).	- Whenever delay in menstruation of 14 days or more or pregnancy are identified at either at bi-monthly follow-up or contact with subject between follow-ups	- Request that subject completes a pregnancy test within a week after detection of menstruation delay if pregnancy is not confirmed (For clinic based model, pregnancy can be tested for at study clinic).	- FA19 to document pregnancy test result
		- Schedule an in-person follow-up within a week after confirming pregnancy (if subject is not in clinic when first confirmed). An in-person follow-up in this case is only required if pregnancy occurs before the end of first year post initiation of supplementation and blood sampling is required for folate measurement.	- FA16 to document folate level
		Obtain blood samples for a folate testing (if applicable).	- FA06 (if pregnancy is confirmed)
		- If pregnancy is confirmed, calculate gestational age and advise subject as when to stop taking the study pills	
		- If subject is not pregnant, then proceed with the regular follow-up and supplementation schedule for non-pregnant subjects	
Prenatal follow-up with subject	Bimonthly follow-up during pregnancy	Contact subject to inquire on pregnancy progress and subject health status	FA07 supplement
Prenatal follow-up with doctor	Monthly follow-up during pregnancy	Contact subject’s prenatal care provider or abstract from prenatal care records to monitor pregnancy events	FA07
Follow-up after miscarriage/stillbirth occurrence	Within 60 days of Miscarriage/Stillbirth	- Check willingness of subject to resume participation in the study	- FA08
		- Contact subject’s doctor (if possible) to obtain information about miscarriage/stillbirth event and material	- FA09&10
		- Check the presence of clefting	- FA13
		- Photographic documentation	
		- Complete study termination form if subject is unwilling to continue in the study	
Follow up after delivery (live birth)	Within 60 days of delivery date	- Check delivery events, maternal health behavior during pregnancy, and infant health measures with study subject; check subject’s readiness to resume enrollment in the study	- FA11
		- Evaluate the infant in person to check for presence of craniofacial or other malformations.	- FA12
		- Complete study termination form if subject is unwilling to continue in the study	- FA21 and FA21 Supplement
		- Contact subject’s doctor (if possible) or review medical records to check the presence of craniofacial malformations including clefting and other defects as well as delivery complications; - Photographic documentation	- FA13
Post-termination follow up	Two months after termination from study	Determine whether the subject became pregnant and request consent to contact her prenatal care provider	- FA14
- Screening and enrollment	After subject agrees to resume enrollment in the study	- Follow standard screening, enrollment procedures listed above	- FA02
- Verification of B12 and pregnancy, dispensing study pills, and Baseline contact		- Subject remains in the original randomization group	- FA03
			- FA04

#### Administration of data forms

Research staff will administer and complete the data collection forms being used for this study using pencil and paper format in primary subject language (Portuguese). Subjects will also self-complete mailed follow-up forms in the outreach model. Research staff will transcribe completed data forms to the study database.

#### Collection of biological samples/shipping

Each blood sample will consist of 4–12 millimeters of peripheral venous blood collected from the subject. Blood will be collected by the Clinical Coordinator or other qualified staff in vacuum tubes either containing anti-coagulant (1.5 ml) or not. All blood collection tubes will be identified by date, name, and subject ID number. Blood samples will be delivered to the laboratory to be analyzed. Blood samples obtained during follow-up outside of the clinic (such as at subject home) will be stored in portable freezers before being delivered to the laboratory.

#### Laboratory analysis

See Section 3

#### Primary and secondary outcome measures

##### Primary outcome measure

The primary outcome assessed is a measure of recurrences of NSCL/P in offspring of the trial mothers. All live born children born of at least 500g or gestation of 24 weeks or beyond with NSCL/P (only Tessier X clefts included) will be identified by case finding and verified by in person clinical exam. For the classification of cleft lip and cleft palate malformations, the following schemata taken from the Division of Plastic Surgery and Burns of the Department of Surgery of the University of Sao Paulo Medical School, Brazil will be used in Table 6.

**Table 6:** Classification of cleft lip and cleft palate malformations:

**Group I**	**Pre-incisor foramen clefts (Clefts lying anterior to the incisor foramen). Clefts of the lip with or without an alveolar cleft.**	
	a) Unilateral	
	Right	Total – when they reach the alveolar arcade
	b) Bilateral	Partial
		1) Total}On one or both sides
		2) Partial
	c) Median	1) Total
		2) Partial
Group II.	Trans – incisor foramen clefts (Clefts of the lip, alveolus and palate)	
	a) Unilateral	Right
		Left
	b) Bilateral	
Group III.	Post-incisor foramen clefts (Clefts lying posterior to the incisor foramen)	
	1) Total	
	2) Partial	
Group IV.	Rare facial clefts	

*Taken from Division of Plastic Surgery and Burns of the Department of Surgery of the University of Sao Paulo Medical School, Brazil (Spina* et al.*, 1972).*

##### Secondary outcome measures

Table 7 lists the secondary outcome measures to be recorded

**Table 7:** Secondary outcome measures

**Secondary outcome**	**Measurement**
Serum/red cell folate levels	Measured using appropriate equipment (e.g. the Elecsys 2010 (Roche) or Immulite 2000) and reported in ng/ml.
Recurrence and severity of CL/P	A severity score will be generated based on Table 6 above and the classification reported in Additional file 1
Occurrence of twinning	Greater than 1 fetus per current pregnancy
Occurrence of a miscarriage/spontaneous abortion	Fetal loss before 20 weeks of pregnancy
Incidence of Preeclampsia	As reported according to local obstetric standards
Occurrence of other birth defects	As diagnosed by clinical exam.
Birth Weight of the baby	As measured at birth (in grams)
Gestational age at birth	As determined by the obstetrician (in weeks-days)

### Training

#### Training study personnel in data collection

The aims and objectives of the study and the study protocol procedures will be provided to all research staff at training. Research staff will be evaluated at training via the use of a posttest. For those who demonstrate lack of proficiency, additional training and mentoring will be provided. Subsequently, individual training will be offered separately.

Clinical Coordinators and other local staff will be trained by the Co- Principal Investigators, core research staff including staff who were involved in the pilot study, and staff from Iowa’s research unit and RTI. Training will include (1) conducting recruitment, interviews, completion of data collection forms; (2) delivery of intervention; (3) blood collection; (4) ethical conduct of research. Future study personnel to be hired for the main study will receive similar training with input from the pilot study staff. In case of turnover, the newly hired staff will also receive similar training. Any drivers hired for the study should hold appropriate driving licenses for participating country. Training in data entry and transmission and in using the DMS will be provided by Data Center (RTI) staff.

#### Job descriptions of study personnel

a) >Principal Investigator: Provides study design guidance, administrative oversight, and budget/finance review for the entire project. His background is in clinical genetics, pediatrics, molecular biology, and the etiology of cleft lip and palate.

Co- Principal Investigator: Will oversee clinical case definition, provide access to subjects through the clinic, develop protocol and MOO, direct the study personnel, be responsible for translations and oversee the laboratory analysis of blood samples.

Study Coordinator (Iowa): Will be responsible for coordination with RTI, Iowa, and the study sites in Brazil. He will be involved in the development of the protocol, MOO and data forms.

1. Clinical Coordinators/Study Nurses (local units—Brazil): Will be responsible for ensuring the proper conduct of the study, maintaining master files of the protocol and the MOO, acting as the liaison between study subjects and the clinic, conducting follow-up of subjects including dispensing the study pills and collection of blood samples, monitoring pregnancies that occur in the subject group, maintaining an inventory of dispensed and stored drugs, distributing and receiving back folic acid pill boxes, keeping the warehouse supplied with all biological material needed, coordinating blood analysis with the clinic laboratory, checking laboratory data (principally to monitor B12 deficiency), printing questionnaires to be administered to study subjects, keying in questionnaire data to the computer, performing the re-keying of a pre-determined percentage of data forms, entering free-text data in the main computer in English, editing all data entry done, and communicating with RTI for correction and transmission of entered data.

b) >Data entry clerk (outreach model only): Keys in data to the DMS. Check the transcription of questionnaire data to computer files. Perform the re-keying of a pre-determined percentage of data forms. Enter free-text data in the main computer in English. Edits all data entry done.

c) >Administrative assistant: Responsible for coordinating correspondence as well meetings with foreign collaborators; and, elaborating and translating all written materials in English (protocols, manuals, informed consent, etc.).

d) >Local IT staff: Responsible for maintaining maintain database, troubleshooting, software and hardware problems, and interface with RTI and data management.

e) >Drivers (outreach model only): Drive study personnel safely and efficiently to their destinations; ensures the safety of the vehicle, passengers and material transported.

f) >Other staff: The clinics may utilize adjunct personnel on an as needed basis such as health science students who are interested in gaining research experience to assist in research and data management activities after receiving proper training.

#### Training materials

Training materials will include the protocol, the manual of operations (MOO), hand-outs, overheads, slides, role-playing, case-discussions, data forms, and phlebotomy equipment (if study staff will be involved in blood collection).

#### Certification of study personnel

All study personnel undergoing training will be certified to work on the study once they have completed the training, passed the certification exam, and satisfactorily mastered the components of the study.

#### Maintenance of training and certification

Maintenance of training will take place at an annual meeting of all staff in which reinforcement of protocols, changes in methods, and problems encountered will be discussed. Refresher training sessions will also be provided during group meetings combing all sites on an as needed basis.

#### Training in ethical issues

All research staff must receive training in ethical and responsible conduct of research. Study personnel can take the English version of the “Human Participants Protection Education for Research Teams” online course, sponsored by the National Institutes of Health ( http://cme.cancer.gov/c01/nih_intro_01.htm). Alternatively, a special ethics training session based on the Research Ethics Training Curriculum created by Family Health International ( http://www.fhi.org) may be substituted. This training is available in Portuguese at the following website: http://www.fhi.org/sp/RH/Training/trainmat/ethicscurr/RETCPo/index.htm.

### Data collection and management

#### Overview

The Data Center maintains the central database for the study and works with the Research Unit and the National Institute of Child Health and Human Development (NICHD) when analyzing and publishing data from the study.

#### Facilities

##### Computer hardware and software

The study clinics will be equipped with desktop personal computers which will be used for entry of questionnaire data, keeping the study pills inventory, counts of unused pills, blood analysis results, and other study files. Desktops will be equipped with data back-up and transmission software.

Data collected by each clinic site will be transmitted to the Data Center over the Internet using software provided by RTI.

##### Laboratory equipment

The laboratory at each hospital with which the clinic is affiliated will be used for analysis of subject blood samples if possible. These laboratories will have to be equipped with appropriate equipment to be able perform measures of folate and B12 levels. In case of inability to do B12 and folate analyses at the clinic laboratory, local laboratories will be identified and contracted to perform the analysis.

#### Data entry

Each study site is responsible for entering and updating all subject records into the study database. The Clinical Coordinator and/or data entry clerk will be primarily responsible for this task. A second keying of a percentage of the completed data forms for verification will be performed. The percentage of the data forms to be re-keyed will be relatively high at the beginning and will start declining as the study progresses due to acquiring greater experience and familiarity with data entry system. The Clinical Coordinator (data entry clerk) will resolve inconsistencies before transmission to the Data Center.

The completed data forms will be entered onto data entry screens in Portuguese. Responses with free text will be translated from the local language into English by the Clinical Coordinator and keyed in English.

#### Data editing and error resolution

The Research Unit, in collaboration with the Data Center, will develop the data entry programs. The data entry programs will include the following edit features:

Field checks (i.e., only numeric data are allowed in numeric fields)

Required data item checks (i.e., data entry cannot proceed until a legitimate value is entered into that field)

Range checks and/or valid value checks

Checks for legitimate data variables

Within form logical consistency checks (e.g., age and date of birth are consistent)

Logical branching algorithms (e.g., the program skips over sections if prompted by a previous entry)

Check digit verification on the ID number to help ensure accuracy

Any restrictions that are overridden during data entry will be documented by the DMS in an audit trail.

On a routine basis, the Research Unit study sites will run edit checks that include the following features:

Eligibility Checks: To check inconsistencies or apparent protocol violations on the eligibility forms.

Date/Gestation Edits: To check inconsistencies between the delivery gestation calculated and other date inconsistencies (e.g., between birth date/time and delivery date/time).

Maternal and Baby Outcome Data: To check inconsistencies between delivery data and data recorded for the neonate.

Screening Log Edits: To check inconsistencies on the screening logs and between the logs and the database

Compliance Data: To check consistency within the compliance and drug dispensing recorded on the study visit forms.

Laboratory Value Checks: To check for unusual or missing lab values on the enrollment form and on the study visit forms.

Missing/Overdue Forms

A special program will be run that checks for serious problems in identification of the subjects, such as duplicate study identification numbers.

As the Data Center receives transmissions from the Research Unit study sites, edit checks similar to those describe above are performed. In addition, further consistency checks are performed. Such checks include: identification of duplicate or missing forms; performing comparisons that require computations or variable coding; and checking values across multiple records.

The Data Center generates and examines the edit check reports. Any edit with a message that explains the problem satisfactorily is marked as OK. The Data Center notifies the Research Unit study site of any unexplained edits or errors and requests clarification.

Any corrections to the database in the study sites are accompanied by notes written directly into the database as part of an audit trail. The initials of the research staff making these changes are automatically attached to the notes.

#### Transmission of data

##### Within country

At each clinic site, the study office will house the Research Unit database for the study on a designated personal desktop computer or a server if available. Study databases will be maintained separate for each study site.

##### To data coordinating center

Data files will be transmitted from the Research Unit offices at the clinic sites to the Data Center (RTI) using the transmission software provided. Transmissions from the server are set to occur routinely. The Data Center will notify the Research Unit when data files that were expected are not received.

#### Security

Research Unit personnel at each study site will keep all completed data forms in a locked facility. Only authorized research personnel will have access to the data forms of the study.

All data files transferred to the Data Center will be stripped of personal identifiers. Any printed material from the data files will be maintained in a locked archive.

The study database will be password protected. Only authorized research personnel will have access to the research database. Data transferred to the Data Center will be password protected, and access will be limited to authorized personnel only.

#### Database construction

The Data Center creates a final database after the completion of the final phase of data collection. The Data Center, in collaboration with the Research Unit, and NIDCR will develop the specifications for this database during the final phase of data collection. The Data Center will deliver one copy of this edited, fully documented database to the Principal Investigator. The data are delivered in a format that will allow ease of import to frequently used statistical packages. If formatting is requested for a particular statistical package, the Data Center will make every effort to satisfy the request.

#### Monitoring data collection

The Data Center will use RTI software to monitor data collection and data processing activities. If data are not received when expected, the Data Center will promptly follow up with the Research Unit. Regular reports (as outlined below) will be produced on the progress of data collection and database development for the use of the Data Center, the Research Unit, and NICHD. These reports will include enrollment reports, forms received, delinquencies, missing and invalid data, and participants due for follow up. The Data Center will also monitor their own data editing efforts to ensure that all failed edits are resolved.

The Data Center will carry out edits that may include

Redoing all the keying edits as a double-check,

Looking for duplicate or missing forms,

Performing comparisons that require computations or variable codes, and

Checking values across multiple records.

#### Reports

The Data Center will collaborate with the Research Unit in the development of any progress reports throughout the course of the study. Periodic reports are expected to be submitted to the DSMB, the Data Center, and NIDCR.

##### Routine reports for local use (e.g., Monitoring reports)

The Research Units at the study sites will generate routine reports for local monitoring. Reports may include enrollment, study visit tracking (scheduling, missed visits and contacts), laboratory results, and missing data forms. The research unit at University of Iowa and the Data Center will work with the local Research Units to create other reports deemed necessary for the proper monitoring of study implementation.

##### Data safety and monitoring board

The Data Safety and Management Board (DSMB) will periodically evaluate the progress of study and the safety and efficacy of the intervention. The Data Center collaborates with the Research Unit to submit to DSMB study reports every 6 months including serious protocol violations and adverse events, on a schedule agreed upon by the DSMB (this is typically once every six months). A detailed description of the responsibilities, organization, and functioning of the DSMB is provided in the Charter for the Data and Safety Monitoring Board for the Oral Cleft Prevention Trial.

##### Data center

Ongoing data reports including progress of subject enrollment are transmitted to Data Center. The reports include subject recruitment, data verification rates, protocol violations, and adverse events. The frequency of data transmission is decided in consultation with the Data Center.

##### NIDCR

The Research Unit will submit an annual progress report to NIDCR and, thus, will provide study and site performance information as stipulated. These annual continuation reports must be submitted 2 months before the end of each project year and will also be sent to the local IRB.

##### Adverse events

The following requirements apply to events involving the mother, the fetus or newborn child, and any other person who may ingest the study medication.

The Co-Principal Investigators or Clinical Coordinators must fax or e-mail an adverse event form in the stated timeframes to the Data Center (please see MOO Section 14.2 Method of Reporting for complete instructions). The Data Center will forward the report to the Principal Investigator, the NIDCR Program Official and Project Officer, and any additional assigned contacts. An adverse report is required for the following events:

All Fatal or Life Threatening events, ***whether or not*** there is any possible association with use of the study medication or participation in the study, must be reported within 24 hours.

Events that are both Serious and Unexpected, ***whether or not*** there is any possible association with the use of the study medication or study participation, must be reported within 7 calendar days.

Other adverse events that are deemed reportable by the clinic staff must be reported within 7 calendar days.

The adverse event reporting form will monitor the following specific events:

Miscarriages

Twinning

Death of mother

Death of infant (if occurring in the neonatal period)

Overdose of pills by mother

Ingestion of pills by anyone other than the mother

Illness in mother requiring hospitalization or medical treatment

Illness of infant (if occurring in the neonatal period)

Occurrence of Preeclampsia.

The Research Unit foreign site IRB will be notified by the Co-Principal Investigator, in accordance with the IRB’s policy for reporting adverse events.

The Data Center will inform the DSMB, at its regular meetings, of any adverse events experienced by study subjects. The Principal Investigator and the Data Center will also notify the IRBs at the University of Iowa and RTI according to the requirements and time frames of these local IRBs.

### Statistical analysis

#### Analysis plan

An intention to treat analysis will be first applied to test the primary and secondary outcomes of the study, where each randomized subject that contributes an informative observation to these outcomes (e.g. a birth) will be included in the analysis, regardless of compliance with study pills or follow-up schedules. Accordingly, randomized subjects will not be purposefully excluded from the study except if they become permanently sterilized or in the unexpected cases where they may no longer take folic acid due to a significant health problem (e.g. allergy to folic acid). The first analysis for the primary hypothesis will exclude subsequent births for subjects who continue to participate in the study after giving birth in the study as these multiple observations will be correlated yet these will be included in secondary analyses that account for multiple observation correlation as further described below.

Due to the randomized design of the study and the large sample sizes, the two treatment groups are expected to be equivalent for factors that may affect recurrence of CL/P and the other secondary outcomes. Therefore, the effects of the treatment dose on study outcomes will first be analyzed using simple bivariate tests such as Fisher’s exact test for rate differences (e.g. CL/P recurrence) and means test for continuous outcomes (e.g. folate levels). The primary estimated effect of the 4 mg folic acid dose compared to 0.4 mg on CL/P recurrence will reflect an average estimate across the two risk groups of women with CL/P and mothers of children with CL/P, but we will also evaluate potential interaction between the dose effect and risk group (such as by using a chi-square test for a common odds ratio across the two groups) and test for the effect of the intervention separately in the two risk groups (though at reduced power).

As secondary analyses, we will check for potential selection bias in the sample of subjects who contribute informative data for analysis (e.g. births or folate levels) due to non-random choices of subjects to participate in the study or to get pregnant and/or give birth (mainly relevant for the primary hypothesis), resulting in informative sample censoring. The issue of concern is not a biased estimate of the intervention effect in the used data samples (randomization solves this problem), but whether these effects are related to any non-randomness in the nature of the used sample. Given the limited availability of data on important characteristics for potential subjects who do not respond to the invitation to participate (refuse to participate, not located, etc.) across the sites, we will analyze dropouts and absence of informative births as potential processes for sample selection bias. We will estimate separate models using logistic regression for propensity to continue enrollment in the study versus dropping out and for giving birth versus dropping out or participation without giving birth. This will be done as a function of characteristics that may affect these propensities such as age, marital status, schooling level, employment status, fertility preferences (child wantedness, contraceptive use), cleft risk group (woman with a cleft or mother to a child with a cleft), previous fertility history (e.g. number of previous children), and area of residence. Data is collected on all these characteristics at enrollment. The propensities will be used as inverse probability weights for the analyses of effects of intervention on study outcomes. The propensities for giving birth will apply to the outcomes measured among study births (cleft recurrence and severity, birthweight, gestational age, etc.) while those for maintaining enrollment will apply to outcomes measured at the subject level (e.g. folate levels). For pregnancy-level outcomes, such as miscarriage, twinning, and pre-eclampsia, propensities for pregnancy (in reference to dropping out or participating without getting pregnant) can be estimated.

Secondary analyses of intervention effects by level of compliance with study pills will be conducted. In addition to the bivariate analyses of intervention effects on study outcomes, we will also employ regression analyses (logistic regression for binary outcomes) that adjust for known and measured risk factors, in order to account for any differences between the two treatment groups due to inadequate randomization, though this is considered unlikely. Several behavioral and environmental factors may affect the recurrence of NSCL/P and will thus be controlled for including smoking, alcohol, age, other vitamin use, age, poverty (instrumented by education, employment), and birth order, in addition to other factors such as the subject risk group (women with cleft or mother to a child with cleft). Interaction effects between the study intervention and these characteristics can be evaluated in these analyses as well. Correlation between subjects recruited at the same sites will be accounted for by either including indicator variables to represent the recruiting site or by using a Generalized Estimating Equations (GEE) or a Hierarchical (Mixed) model, which also can be used to account for within-subject correlation in case of multiple observations per subject.

#### Power analysis

Table 8 presents estimates of prospective number of births in the two study groups required for 70 and 80% statistical power and 30, 50, and 70% reductions in CL/P recurrence for alternative recurrence rates of 3, 5, and 7% in the 0.4 mg group. The 30% reduction represents a lower effect that is still very clinically significant, while the 70% represents an effect similar to that observed for NTD recurrence [118]. The recurrence rates cover most of those reported in the literature and we use the 5% rate as a primary reference for our total suggested sample size given that it represents a proportional estimate of the 4 and 7% recurrence rates assuming that 30% of the study subjects will be women with clefts and 70% will be mothers to children with clefts. The power calculations are based on a Type I error probability of 5%. We use a one sided test as the recurrence rate in the 4 mg is not expected to exceed that of the 0.4 mg group given the converging evidence of protective effects of folic acid; a Fisher’s exact test is used.

**Table 8:** Power analysis for primary hypothesis

**Baseline recurrence rate**	**% Reduction**	**Power**	**Total births**	**Total subject years**
*0.07*	70	0.8	524	5475
		0.7	418	4368
	50	0.8	1114	11641
		0.7	874	9133
	30	0.8	3324	34734
		0.7	2574	26897
*0.05*	70	0.8	742	7753
		0.7	590	6165
	50	0.8	1582	16531
		0.7	1240	12957
	30	0.8	4734	49467
		0.7	3666	38307
*0.03*	70	0.8	1254	13103
		0.7	996	10408
	50	0.8	2676	27962
		0.7	2096	21902
	30	0.8	8024	83845
		0.7	6210	64890

For a 50% reduction in a baseline rate of 5% recurrence, a total of 1582 births distributed equally across the two treatment groups are required. This number of births also provides acceptable power (0.75) to detect a 60% decrease in a 3% baseline recurrence rate, or a 40% decrease in a 7% baseline risk. If the 4 mg dose shows a preventive effect similar to that seen for NTDs (70%), the required numbers of births are halved regardless of the baseline recurrence risk (range from 524 to 1254 births for 80% power). The numbers of births required to observe a 30% reduction are about three times as large those for a 50% reduction.

This study may also allow evaluating secondary outcomes. The effect of the intervention on severity of clefting will be assessed. We will also stratify our analysis to examine differences in outcomes in the women born with CL/P independently from women who have a child with CL/P. The data from previous studies suggest a similar affect of folic acid supplementation in mothers with CL/P when compared to mothers who had a child with CL/P but as described below we will examine the groups together and separately.

Table 9 lists a few plausible effects and changes between the 4 and 0.4 mg groups and the total number of births needed to observe those effects with 80% power (type I error of 5%). Each of these (and others) will be studied as secondary outcomes. The DSMB will also monitor these outcomes to determine if significant untoward side effects are occurring (increases in miscarriage rates for example) but this is done independently of the research staff. All power simulations are based on a two-sided test except for the severity measure which is based on a one-sided test given that severity is expected to decrease with the higher dose.

**Table 9:** Power to evaluate secondary outcomes

**Secondary outcome**	**Total births**
Birthweight (difference of 100 gm)*	928
Birthweight (difference of 200 gm)*	234
Gestational age (difference of 2 days*	2484
Gestational age (difference of 4 days*	622
Pre-eclampsia-Change by 40% from 10% baseline rate	1540
Pre-eclampsia-Change by 50% from 10% baseline rate	948
Severity (50% change from cleft lip with palate to cleft lip only in affected cases with 25% overall cleft prevention)	680
Miscarriage (30% change from 15% baseline rate)	1629
Miscarriage (50% change from 15% baseline rate)	562
* Assumes SD of 543 grams (birthweight) and 17.8 days (gestational age) as estimated in our studies in these populations.	

#### Sample size

In order to estimate the numbers of subjects and subject participation years required to obtain these numbers of births given that subjects are enrolled at pre-conception, we use a birth rate of 0.0957 births per subject-year, which is the rate observed to date in the ongoing study. Subject participation years are included in the fifth column of Table 8.

For the primary hypothesis, a total of about 16,531 subject-years are potentially required to provide the 1,582 births. Doubling the birth rate (0.19) would decrease the number of required subject years for all scenarios by half; about 8,266 total subject years will be needed to obtain 1,582 births. We believe the 0.0957 rate is conservative and underestimates the birth rate likely to be observed in this study due to the short period on which it is based and given that at least half of the subjects enrolled to date have expressed a desire to have (more) children. Any increase in the birth rate will result in a proportional decrease in the total required subject years.

Our estimate of the targeted sample size of subjects uses the estimate of 16,531 subject participation years as the lower bound for targeted subject years and takes into account the recruitment capacities of the participating sites. We will enroll about 21 subjects per week across the 4 sites using the clinic-based model (6 subjects in each of HRAC, Recife, and Salvador, and 3 in Porto Alegre) during the first four years, which are expected to provide about 12,035 subject-years, and will enroll about 1000 subjects by the end of the first year using the outreach model at the Bauru site, which are expected to provide about 4,250 subject years. Follow-up of subjects is scheduled to be completed about nine months after the end of recruitment. The 550 subjects currently participating in the study at Porto Alegre, Salvador, and Bauru are expected to add about 2,612 subject years if they maintain their participation in the continued phase. This is independent of the number of births that this group already had (50 births) which by itself lowers the sample size (1582 births) and the subject years required for the continued phase. Therefore, a total of about 18,897 “birth risk” years are targeted in the continued phase to ensure that the minimum estimate of 16,531 subject years is reached. About 5,368 new subjects are thus expected to be enrolled in the study including 1,000 subjects at the Bauru site using the outreach model and 4,368 subjects across the study clinics using the clinic-based model (1,092 subjects per year). Table 10 provides a summary of new subject recruitment and subject years.

**Table 10:** Estimates of new enrollment and subject years

**Site**	**Total of new enrollees**	**Total subject years from new recruitment**
Bauru – Outreach Model	1000	4250
Bauru – HRAC clinic	1248	3439
Salvador	1248	3439
Recife	1248	3439
Porto Alegre	624	1719
*Total*	*5368*	*16281*

Note: The table shows the total number of new subjects estimated to be enrolled by March 2011 and the total subject years estimated to be provided by this recruitment by December 2011.

#### Interim analysis and study monitoring

The DCC will conduct analyses of screening, recruitment, and participation rates as well as analysis of compliance, every quarter of the study. The DCC will compare screening, recruitment, participation, and compliance to the projected rates. If the observed rates are below the expected rates by 20% for any study site, the study team will examine the causes of the discrepancy and develop actions to improve these rates. The DCC will examine the characteristics of dropouts and non-compliers with study procedures and reasons for non-participation when possible. For data quality, error rates will be reviewed from double keying. The DCC will check lab data for consistency of collected information. The birth rates among study subjects will be estimated periodically to determine the number of subjects to be recruited. The DCC will also monitor the generate reports on study adherence and adverse events. A summary monitoring report will be provided to the Data Safety and Monitoring Board (DSMB) for their semiannual meetings.

To examine the efficacy of the intervention, we propose two interim analyses at 40% and 70% of the targeted number of births and a final analysis. The corresponding alpha boundaries are 0.002, 0.017, and 0.031 for a one sided test and unequal spacing of analyses using O’Brien-Fleming spending boundaries [123]. For the primary hypothesis of this study, the power remains at approximately 80% and the targeted sample size remains sufficient to allow the proposed interim analysis.

All interim analysis reports will be provided to the DSMB for their semiannual meetings. Data will be provided on all the monitored adverse events that are listed in the protocol. The DSMB will review the adverse events by treatment groups, the observed enrollment rate, and the required number of subjects based on observed birth rate at each meeting and make decisions about study continuation. The DSMB will review the rates of primary outcome by treatment groups at each interim look and make decisions about study continuation. The magnitude of any observed rates of primary outcome differences by treatment status and what to consider a "substantial" difference will be revisited at each interim looks.

In addition, conditional power/stochastic curtailment method [124] may be used if requested by the DSMB due to small observed differences in primary outcome rates between treatments. This analysis involves calculating the conditional probability of rejecting the null hypothesis given a specific alternative and the sample size at the time of the analysis. A very small probability suggests that the null effect may be inevitable.

Sample size and spending boundary estimations were performed using the East version: 4.0.1.50 by Cytel Software Corporation.

### Quality control

The quality control plan will involve establishing a competitive set of selection criteria of study personnel, adequate training and certification of training, adequate on-site monitoring of study progress, periodic and on as-needed basis conduction of site visits by principal investigator, NICHD Staff Science Collaborator, and the Data Center, and appropriate provision of guidance and feedback to study personnel upon occurrence of protocol violations. The quality control process also includes the following: verification of data entry, establishing a toll-free phone line for subjects to call for questions, monitoring quality of folic acid pills, providing adequate and continuous guidance to subjects regarding proper intake and handling of pills, and continuous and adequate reporting of adverse events and protocol violations.

#### Selection of study personnel

Selection criteria to be utilized in the recruitment and hiring of study personnel include (1) appropriate educational level, (2) high technical competence, (3) good communication skills, (4) a commitment to and interest in the study design, and (5) quality related professional experience.

#### Job descriptions

Please see Section for job descriptions. The table below lists selection criteria for each position.

#### Training procedures

All study personnel will receive training as outlined in Table 11.

**Table 11:** Staff selection criteria

**Job title**	**Selection criteria**
***Brazil Research Unit Staff***	
Clinical Coordinator (or Study Nurse)	Higher or intermediate schooling level*, high communication skills that allow for conducting successful interviews with subjects, quality skills in drawing blood samples (if needed), as well as acceptable computer knowledge/familiarity that enable them to manage laboratory and patient compliance data.
Data entry person (outreach model only)	Intermediate schooling level*, good skills in using basic computer packages such as Word and/or Excel, as well an acceptable speed of data entry
Drivers (outreach model only)	Fundamental schooling level*, having a driver’s license and showing a mature attitude and responsibility towards driving.
Administrative Assistant	Higher schooling level*, high communication skills that allow coordination of correspondence and meetings with the Principal Investigator and other related personnel, as well as having a very good knowledge of written and spoken English to be able to translate both written and spoken English to Portuguese and vice versa.
***US Staff***	
Study Coordinator	PhD or equivalent degree in a health care related field, good experience in project management and research conduct, as well as strong communication skills and commitment to responsible care.

*The Brazilian Educational System is divided into three levels: fundamental, intermediate and higher education. Fundamental school extends over eight grades. Intermediate school extends over at least three grades, and may include professional training. It thus may last for four or five years and provides professional qualification. Higher education is composed of two different levels: undergraduate (e.g., full-nurse) and graduate. Additionally, professional training may be provided to students who have only completed fundamental school.

#### Certification procedures

All the study personnel will undergo training before data collection commences. The competence of the trainees will be certified at the end of the course and via a post-training test. The Co-Principal Investigators will be responsible for certification.

#### On-site monitoring

At each site, the research will meet on a periodic basis as determined locally to review ongoing activities and assurance of quality control.

#### Site visits

Site visits will be conducted by the Principal Investigator, NIDCR, and the Data Coordinating Center to monitor the quality of study implementation by the Research Units. Site visits will be conducted at least once a year and on an as needed basis (if there are study implementation concerns that seem to require immediate evaluation and/or intervention), in consultation with the local IRBs and will include the participation of the Principal Investigator, NIDCR Program Official and/or Project Officer, and the Data.

Coordinating Center Principal Investigator or his/her designee. The NIDCR, the Data Coordinating Center, and the U.S. and foreign Research Units will collaborate in the planning and implementation of each site visit.

The purpose of a site visit is to monitor study procedures including those related to the protection of human subjects, recruitment and eligibility, data collection and management, the protocol violations, and adverse event reporting.

Methods for monitoring include chart reviews, interviews with research staff, and facility observation. The site visit will include a feedback session to review results and suggest recommendations for improvement. The Data Coordinating Center will submit a report to NIDCR that documents the assessment, results, and feedback given to the Research Units, as well as its recommendations.

#### Feedback for protocol violations

The Co-Principal Investigators will provide guidance and feedback to the appropriate study personnel on any protocol violations. A list of these is provided in Section 4.6. In some cases, retraining or retesting may be indicated. Protocol violations will also be reviewed at weekly staff meetings.

#### Quality control of study pills and randomization

The folic acid pills for the ongoing study are manufactured by ATIVUS Pharmaceutical Industries in Brazil ( http://www.ativus.com.br/). The manufacturer will conduct quality control tests for each batch of pills including identification test of the raw folic acid material used in the pills (prior to production), assay tests for the folic acid dosage in the pills, content uniformity tests (to ensure that the dosage is consistent across pills), and dissolution tests to ensure that the pills dissolve adequately in a standard time. The manufacturer will provide these results to the investigators before labeling and shipping the pills to the sites. If the results are not within the acceptable range, the manufacturer will reproduce and retest new batches of pills. Only batches with documented test results that are within acceptable ranges will be shipped to the sites.

The manufacturer will label the blister packs with lot numbers, enclose the blister packs in boxes, and place the 0.4 and 4 mg boxes in separate containers. An independent contractor will label the pillboxes, at the manufacturer’s facility, with the serial numbers that are matched to randomization assignment. The labeling will be conducted under RTI’s supervision (see section 4.1.1). The accuracy of the labeling will be independently tested, before sending the pills to the sites, through randomly selecting and sending to RTI a sample of boxes that are labeled with the serial numbers. RTI will compare the lot numbers on the blister packs to the serial numbers on the pillboxes to ensure that serial numbers and lot numbers match correctly. Further, the pills from this sample will be sent to an independent laboratory in the US to conduct assay content tests to ensure that the folic acid dose matches the treatment assignment of the lot numbers (0.4 versus 4 mg). These steps will identify errors in labeling the blister packs and in labeling with serial numbers. The latter can result due to incorrect packaging the pillboxes into the 0.4 and 4mg containers by the manufacturer or due to inaccurate serial number labeling by the contractor.

Different procedures will be used to address labeling errors depending on the error type. Errors in serial number labeling will require verifying the lot numbers inside each pillbox to ensure that they match the assigned serial number and relabeling those that do not match. All the pillboxes will be rechecked when this error occurs. Errors in blister pack labeling will require production and labeling of new pills, which will be test using the procedures described above.

Pills will not be sent to the sites before all the quality control procedures are completed. This necessitates that pill orders are placed at least 5 months before the intended date of shipping to the sites.

### Sustainability

#### Dissemination plans for research findings

Significant findings from the analyzed data of either the primary or secondary hypotheses will be discussed by the investigators. When confirmed, the data will be presented at national and international meetings in abstract or meeting report formats. Formal publications will be developed from these findings and submitted to appropriate peer reviewed journals. Methodologies such as study design may also be reported.

The ability to obtain high dose (4 mg) folic acid pills at reasonable costs will allow women who are at risk of having a child with NSCL/P, and who are concerned about preventing such recurrence, to benefit from the outcome of this medication, when demonstrated, without facing a financial burden that may hinder this process. In fact, it is estimated that the cost of obtaining high dose folic acid pills by a woman per year falls in the range of 3 to 4 U.S. dollars, which implies that even women who suffer from real poverty may be able to purchase the medications. However, in cases of extreme poverty, where women cannot afford to get folic acid even at such a low cost, local governments and private health organizations (such as the participating clinics) will be highly encouraged to provide the medication to those women at no cost.

#### Processes and equipment within country

Equipment will be retained by the host site including computer hardware and software. Any other purchased equipment will be retained at the host site as well.

#### Plans for use of trained personnel

The Clinical Coordinators participating in the study may be retained in a formal program aimed at prevention if the primary hypothesis is realized. Funding for continued support will come from existing mechanisms of care provided to families of children with clefts including federal and private funding. When continuing research questions are under evaluation, grant support will be sought to provide an ongoing base for investigative work. A national program of vitamin supplementation will be encouraged and sought after the study is completed if results suggest it is warranted.

### Study organization

#### Duties of the research unit

The Research Unit, which consists of a joint collaboration between the local research units and University of Iowa research teams, will be responsible for the following:

1. Initial and ongoing training and certification of the study personnel as outlined in Section 6;

2. Maintaining compliance and follow up rates of at least 80 percent;

3. Disseminating and applying research findings to benefit the study populations, including providing information about related plans on an annual basis (see Section 10.1);

4. Implementing the study protocol and MOO;

5. Managing the process of ongoing reporting to the IRBs, NIDCR, and RTI;

6. Processing and storing the samples collected in the field at each maternal visit, including preparation for folate and storage of unused samples to provide a long-term resource for future studies;

7. Maintaining a database of samples with tracking information and the ability to link with demographic and clinical data; and

8. Analyzing the processed samples for vitamin (serum or red cell folate, B12) status.

The Research Unit is also responsible for other unlisted duties that may be mentioned in the Terms of Award.

The U.S. unit provides oversight, assistance in writing, budget management, and advice on study design.

The Principal Investigator is responsible for the following:

1. Providing the study protocol and other documents as listed in the Terms of Award;

2. Submitting to NIDCR and the Data Center documentation of current approval and the comments of the IRBs of local research unit and the University of Iowa;

3. Informing NIDCR Program Official and Project Officer and the Data Center of all major changes in the status of the ongoing protocol, informed consent, or IRB approval within specific timeframes as indicated in the Terms of Award;

4. Submitting annual progress reports to NIDCR for evaluating the Research Unit Performance; and,

5. Reporting serious adverse events to the Data Center, and NIDCR Program Official and Project Officer and any additional assigned contacts, within specific timeframes (see Section 7.9.6).

A chart of the lines of authority of the staff of the study is provided in Additional file 1.

#### Duties of the data coordination center

Responsibilities of the Data Center (RTI) include the following:

Provide advice on study design, data collection, data analysis, and publication development.

Prepare operations manuals and data collection forms.

Compile monthly and quarterly subject enrollment reports, meeting summaries, progress reports, and other reports as needed.

Design and manage study databases; Assure maintenance of high quality databases, supervise all data collection procedures, and arrange for the most efficient transfer of study data where indicated.

Ensure full compliance with NIH regulatory requirements, including informed consent, reporting of adverse events, human subject safety and welfare provisions, and the requirements of international collaboration.

Provide training to all Research Unit site personnel as needed on data management and analysis, and quality control and quality assurance.

In coordination with the NIH cosponsors, provide periodic on-site monitoring to the Research Units for those studies being performed at that site.

#### Duties of NIDCR

The NIDCR Program Official and Project Officer serve as the principal representatives of NIH, and will provide overall programmatic oversight, coordination, and assistance.

Specific responsibilities include:

•Oversee site participation and performance with the support of the Data Center.

•Participate in study design, data analysis, interpretation, and publication of study results.

### Human subjects

#### Description of participants

Potential study participants include all women attending or registered in the participating craniofacial clinics, for their own craniofacial treatment (when they have NSCL/P) or the treatment of their child with NSCL/P, who meet the inclusion criteria as described in 3.2.1 and who do not meet exclusion criteria as described in 3.2.2.

#### Recruitment

Subjects include women with NSCL/P or mothers of children with NSCL/P who are currently receiving or have received in the past care at the participating clinics, are eligible, and provide consent to participate in the study. Women who are married to men with NSCL/P and at the same time do not fit in the above two groups are not included as a group because of concern about the staff’s ability to convince the women to take folic acid in this situation. Therefore, it is expected that compliance with taking folic acid will be achieved best in women who are included in the above two groups.

#### Informed consent

Informed consent will be obtained from all subjects. The benefits and possible adverse effects of the intervention will be explained to subjects. Subjects will be encouraged to seek clarification regarding the intervention, and efforts will be made to ensure that the information was comprehended. The Clinical Coordinators will read the informed consents to all subjects, and confirmation will be obtained via a signature for literate subjects, and via thumb impression in the presence of a witness for illiterate subjects. Potential subjects will be assured that refusal to participate in the study will in no way affect further treatment or care. A copy of the consent form being used for this study is provided in Additional file 1.

#### Incentives and other benefits

See section 3.8.

#### Cultural issues

This is both a high-risk and a motivated population. There is a risk that during the course of the study families will be found with specific causes of clefts that will change their recurrence risk from what was previously known and that might have relevance for other family members not in the study. This can be dealt with through the usual process of genetic counseling available through the study clinics.

#### Reporting to local IRBs

Each year an annual report of activities undertaken will be prepared. These reports will also include any modification in the original protocol. Reports will be sent to all entities involved in the project, including the local IRBs.

### Publications and presentations

#### Review process

Prior to their submission or application for presentation, all manuscripts, posters, or oral presentations, or other reports of the outcomes of this research effort will be approved by a majority of the senior study staff including (1) the Principal Investigator, (2) the Co-Principal Investigators, (3) the NIDCR Project Officer, and (4) a Senior research officer from the Data Center.

#### Authorship

The authorship of manuscripts, poster or oral presentations, or other reports of the results of this study will be guided by the criteria for authorship formulated by the International Committee of Medical Journal Editors and published in its Uniform Requirements for Manuscripts Submitted to Biomedical Journals (updated October, 2001; available at: http://www.icmje.org). According to these criteria, each author should have participated sufficiently in the work to take public responsibility for the content. Authorship credit should be based only on substantial contributions to (a) conception and design or analysis and interpretation of data; and, to (b) drafting the article or revising it critically for important intellectual content; and, on (c) final approval of the version to be published. Conditions (a), (b), and (c) must all be met.

As a multicenter study, up to twelve authors, as permitted by journals such as the New England Journal of Medicine and the American Journal of Obstetrics and Gynecology, may be identified for a given manuscript, but only those authors fulfilling the criteria above are eligible. As a general, but not absolute, rule, at least one individual from the NIDCR, one individual from the Data Center, the Principal Investigator and the Co-Principal Investigators will be authors for all publications that result from this research.

## Discussion

The costs related to oral clefts are high, and there is converging research evidence of long term psychological and socio-economic effects [125]. Since it is estimated that about 1000 cases of CL/P born each year in the US to women with CL/P or mothers of a child with CL/P, demonstration that a folic acid dose of 4 mg per day results in a 50% reduction could prevent CL/P from occurring in 500 births in the US per year. Application of the strategy in all developed countries would eliminate CL/P in 1,500 children and if applied worldwide roughly 10,000 births a year would be free of CL/P. In the US, the economic costs of oral clefts (both direct and indirect) have been estimated to be about $200,000 per case in present dollars [126], while the cost of folic acid is pennies per day. This study provides an opportunity for huge savings in not only money but the overall quality of life. If supplementation with high dosage folic acid proved effective, it is estimated that supplementation of all at-risk women who would give birth in a particular year in the United States would cost $132,000, compared to avoided costs of about $100 million dollars.

This study will also provide an infrastructure to assess the effectiveness of the proposed folic acid intervention in subgroups of women of different genetic characteristics and risks, particularly in folate metabolism genes. The proposed study will estimate the average treatment effect of 4 mg folic acid supplementation compared to the 0.4 dose, with the gene interaction studies to further check for potential mediation of the effect by genotype. This may help establish more specific clinical guidelines for oral cleft prevention so that the intervention can be better tailored for at-risk women. In this proposed clinical trial, we intend to collect and store DNA samples for each participating subject, but we will not request a specific budget in this grant for DNA analysis. We will be able to conduct DNA analysis and implement the gene-interaction studies under the auspices of other ongoing research support.

## Abbreviations

ANVISA: Agência Nacional de Vigilância Sanitária; CI: Confidence interval; CLO: Cleft lip only; CLP: Cleft lip with cleft palate; CL/P: Cleft lip with or without cleft palate; CONEP: National Committee of Research Ethics; CPO: Cleft palate only; DSMB: Data Safety and Monitoring Board; GEE: Generalized Estimating Equations; FA: Folic Acid; NS: Non-syndromic; HRAC: Hospital de Reabilitação de Anomalias Craniofaciais; HCPA: Hospital de Clínicas de Porto Alegre; IMIP: Instituto Materno Infantil Prof. Fernando Figueira; LMP: Last Menstrual Period; MOO: Manual of Operations; NIDCR: National Institute of Dental and Craniofacial Research; NICHD: National Institute of Child Health and Human Development; NSCL/P: Non-syndromic cleft lip with or without cleft palate; NTDs: Neural Tube Defects; OSID: Obras Sociais Irmã Dulce; RTI: RTI International.

## Competing interests

None of the authors has any competing interests in this work.

## Authors’ contributions

JCM and DMF conceived and designed the study. GW co-designed the study, developed study measures and procedures, and contributed to designing the statistical analyses. ARC, TF, and CP and FQ and CVG contributed to designing the study procedures and data questionnaires. NG contributed to designing and writing the study procedures. THartwell and HC contributed to designing the statistical analyses. Mr. SL contributed to designing and writing the data management procedures. LJ and Atkinson contributed to designing the study. All authors read and approved the final manuscript.

## Pre-publication history

The pre-publication history for this paper can be accessed here:

http://www.biomedcentral.com/1471-2431/12/184/prepub

## Supplementary Material

Additional file 1 Appendix. Click here for file
