# Supplementary material for: Oral cleft prevention program (OCPP)
Source: BMC Pediatr. 2012 Nov 26;12:184. doi: 10.1186/1471-2431-12-184 (PMC3532199; doi:10.1186/1471-2431-12-184)
Supplement: Additional file 1 — Appendix. [file 1471-2431-12-184-S1.doc]

APPENDIX A. CONSENT FORM

**Informed Consent Form**

We are inviting you to participate in the research project of ”Name of the clinic” entitled “Oral Clefts Prevention Program” that aims at assessing the effect of folic acid on the recurrence of oral clefts. This project is aimed at the prevention of oral clefts and is being conducted in Brazilian centers in collaboration with the University of Iowa, the National Institute of Dental and Craniofacial Research (NIDCR) in the United States and RTI International, with funding from NIDCR.

Approximately 6000 women are expected to participate in this study.

We would like you to know that:

 Your participation is voluntary.

 Your decision whether or not to participate will not affect care or your current or future treatment at ”Name of the clinic”.

 You are free to discontinue participation in this study any time and no penalty will be imposed on you or your child.

If you decide to participate, we would like to let you know that:

a. You will have the same chance of being assigned to one of two groups: one receiving 4.0 mg of folic acid or another receiving 0.4 mg of folic acid. You will not be told of your group assignment and the person that gives you the pills will not know. The pills dispensed to both groups will be identical in appearance.

b. Every two months, you will receive 70 pills of folic acid (vitamin) and will be contacted to check on your health. You will be asked to take 1 pill a day. If you become pregnant, you will be asked to stop taking the folic acid pill after the first three months. We will maintain our bimonthly contact with you during your pregnancy. After you complete your pregnancy, you may continue to participate in the study and take folic acid until you become pregnant again or the study is completed.

c. Folic acid is a vitamin, that is, it is a nutritional supplement, not a medicine. It causes no side effects. Folic acid is known to prevent certain birth defects, but may hide the signs of health problems related to vitamin B12 deficiency. In this research, this effect will be closely monitored.

d. 4–12 ml of blood (less than 1 tablespoon) will be collected at enrollment, at pregnancy or the end of the first year, whichever occurs first, and every year thereafter until the end of the study. The blood will be analyzed for concentration levels of folate and of vitamin B12 and for whole blood count.

e. From your blood samples, we intend to extract DNA (genetic material) in order to establish a DNA bank specifically for this study. This will not be done unless you consent.

f. With regard to research or analyses that may be performed using your DNA stored in the study bank, you will be informed and your consent will be requested

g. Blood collection may cause some physical discomfort and a purple stain (hematoma) may occur in the area (this is considered normal).

h. We may contact your health care providers to obtain information on your health status and pregnancy progress and outcomes if you become pregnant. This information will be kept confidential and will only be accessed by research staff.

i. The results of this study will not bring you or your family any immediate beneficial effect. The results will take years to be available. As soon as results are available, they will be presented to you at routine clinic appointments or by mail.

j. The results will be published in scientific journals that are read by health professionals interested in this field.

k. Whenever scientific papers are published, your identity as well as the identity of your child (if you conceive one) will not be disclosed. Neither your name nor the name of your child will be disclosed.

l. All blood test results will be made available upon request and when your participation in the study ends. They will be kept in the general archives of the clinic. The research staff will deny access to them unless written consent from the family, or qualified professional, is provided.

m. The research staff can stop your participation if you experience an allergic reaction to the folic acid pills, have vitamin B12 deficiency or have other problems that might present a health risk for you. If new findings are discovered during the study that may influence your willingness to continue participating, the research staff will notify you.

n. This research may help people in the future. You will be helping us to improve our understanding about the prevention of oral clefts. You may be contacted in the future for participation in research related to this study.

o. Upon enrollment into the study and at every third follow-up completed (i.e. about every 6 months), you will receive one food voucher. The study will cover your transportation costs to the clinic for the study purposes. You will be provided with pre-stamped mailing envelopes when needed that you will use to mail unused study pills to the clinic as instructed.

You may ask about and be informed about the findings of this research. Only authorized people from the research team will be able to review your medical records and use or see any study information that has been collected. You may choose to stop participating in this research at any time and may have all data related to your family erased and/or destroyed by contacting Dr. ”Name of Co-Principal Investigator” at telephone number ”Telephone number”.

If you have questions regarding the research study, you may contact ”Name of Co-Investigator” at ”Telephone number”. Also, if you have any questions regarding rights as a research subject, you may contact _________________________ at telephone number _____________.

I, ,consent to participate in the research project “Oral Clefts Prevention Program”. The study has been fully explained to me and I am aware of all the items described above. My participation is voluntary and herein consented.

With respect to the DNA bank for this study, I

⁭ consent that my DNA sample is stored

!--

| ⁭ do not consent that my DNA sample is stored |  | |  | |
| --- | --- | --- | --- | --- |
| Research Subject Signature |  | | Place Date | |
|  | |  | |  |
| Legal Representative/Parent Signature (or thumbprint) ****Required if the research subject is minor*** | |  | | Place Date |
|  |  | |  | |
| Signature of Investigator who obtained consent (Legible signature, or stamp and signature) |  | | Place Date | |

I state that I was present when this informed consent was read to the research subject and that all of her questions and the nature of the study were adequately clarified.

|  |  |  |
| --- | --- | --- |
| Signature of witness |  | Place Date |

*Two copies of this term are being signed. One to be given to the subject and the other to be kept in the archives of the Oral Cleft Prevention Program

Name of the site Co-investigator:

Phone:

Address:

APPENDIX B. CODES FOR CLASSIFICATION OF CLEFT LIP AND PALATE

Microform right cleft lip 1 Bilateral lip, and total alveolus 15

Right lip only 2 Bilateral lip, right partial alveolus/left total alveolus 16

Right lip and partial alveolus 3 Bilateral lip, right total alveolus/left partial alveolus 17

Right lip and total alveolus 4 Bilateral lip, alveolus, palate 18

Right lip, alveolus, palate 5 Submucous cleft palate 19

Right lip and palate 6 Bifid uvula 20

Microform left cleft lip 7 Cleft palate/ soft 21

Left lip only 8 Cleft palate hard and soft 22

Left lip and partial alveolus 9 Cleft palate hard 23

Left lip and total alveolus 10 Midline cleft lip only 24

Left lip and palate 11 Midline cleft lip and partial alveolus 25

Microform bilateral cleft lip 12 Midline cleft lip and total alveolus 26

Bilateral lip only 13 Midline cleft lip, alveolus and palate 27

Bilateral lip, and partial alveolus 14 Other (describe, including lateral facial clefts) 28

APPENDIX C. LINES OF AUTHORITY OF STAFF

Data Safety and Monitoring Board

NIDCR Program Official and Project Officer

Study Coordinator

Data Center

Principal Investigator

Foreign Principal Investigators

Research unit staff (Clinical coordinators, data entry personnel, drivers)
